# Racial and ethnic disparities in preterm birth: a mediation analysis incorporating mixtures of polybrominated diphenyl ethers

**DOI:** 10.3389/frph.2023.1285444

**Published:** 2024-01-08

**Authors:** Zifan Wang, Cuilin Zhang, Paige L. Williams, Andrea Bellavia, Blair J. Wylie, Kurunthachalam Kannan, Michael S. Bloom, Kelly J. Hunt, Tamarra James-Todd

**Affiliations:** ^1^ Department of Environmental Health, Harvard T.H. Chan School of Public Health, Boston, MA, United States; ^2^ Global Center for Asian Women’s Health, Bia-Echo Asia Centre for Reproductive Longevity & Equality (ACRLE), NUS Yong Loo Lin School of Medicine, National University of Singapore, Singapore, Singapore; ^3^ Department of Obstetrics & Gynecology, Yong Loo Lin School of Medicine, National University of Singapore, Singapore, Singapore; ^4^ Department of Biostatistics, Harvard T.H. Chan School of Public Health, Boston, MA, United States; ^5^ Department of Epidemiology, Harvard T.H. Chan School of Public Health, Boston, MA, United States; ^6^ Department of Obstetrics and Gynecology, Columbia University Vagelos College of Physicians and Surgeons, New York, NY, United States; ^7^ New York State Department of Health, Wadsworth Center, Albany, NY, United States; ^8^ Department of Global and Community Health, George Mason University, Fairfax, VA, United States; ^9^ Department of Public Health Sciences, Medical University of South Carolina, Charleston, SC, United States

**Keywords:** health disparities, race and ethnicity, preterm birth, gestational age, chemical stressors, polybrominated diphenyl ethers, mediation analysis, environmental mixtures

## Abstract

**Background:**

Racial and ethnic disparities persist in preterm birth (PTB) and gestational age (GA) at delivery in the United States. It remains unclear whether exposure to environmental chemicals contributes to these disparities.

**Objectives:**

We applied recent methodologies incorporating environmental mixtures as mediators in causal mediation analysis to examine whether racial and ethnic disparities in GA at delivery and PTB may be partially explained by exposures to polybrominated diphenyl ethers (PBDEs), a class of chemicals used as flame retardants in the United States.

**Methods:**

Data from a multiracial/ethnic US cohort of 2008 individuals with low-risk singleton pregnancies were utilized, with plasma PBDE concentrations measured during early pregnancy. We performed mediation analyses incorporating three forms of mediators: (1) reducing all PBDEs to a weighted index, (2) selecting a PBDE congener, or (3) including all congeners simultaneously as multiple mediators, to evaluate whether PBDEs may contribute to the racial and ethnic disparities in PTB and GA at delivery, adjusted for potential confounders.

**Results:**

Among the 2008 participants, 552 self-identified as non-Hispanic White, 504 self-identified as non-Hispanic Black, 568 self-identified as Hispanic, and 384 self-identified as Asian/Pacific Islander. The non-Hispanic Black individuals had the highest mean ∑PBDEs, the shortest mean GA at delivery, and the highest rate of PTB. Overall, the difference in GA at delivery comparing non-Hispanic Black to non-Hispanic White women was −0.30 (95% CI: −0.54, −0.05) weeks. This disparity reduced to −0.23 (95% CI: −0.49, 0.02) and −0.18 (95% CI: −0.46, 0.10) weeks if fixing everyone's weighted index of PBDEs to the median and the 25th percentile levels, respectively. The proportion of disparity mediated by the weighted index of PBDEs was 11.8%. No statistically significant mediation was found for PTB, other forms of mediator(s), or other racial and ethnic groups.

**Conclusion:**

PBDE mixtures may partially mediate the Black vs. White disparity in GA at delivery. While further validations are needed, lowering the PBDEs at the population level might help reduce this disparity.

## 1 Introduction

Preterm birth (PTB) affects 9%–10% of pregnancies in the United States, and is associated with increased risk of maternal and neonatal morbidity and mortality (1, 2). There are pronounced racial and ethnic disparities in PTB in the United States, with rates disproportionately higher in non-Hispanic Black women than non-Hispanic White women (14% vs. 9%) (3, 4). These disparities may further contribute to higher infant mortality (4) among non-Hispanic Black relative to non-Hispanic White infants. For other groups, studies showed no significant difference in PTB rate comparing Asian or Hispanic women to White women, although the risk appeared higher in certain Asian subgroups (5). Therefore, identifying the potentially modifiable risk factors of PTB, especially those that are unevenly distributed across racial and ethnic groups, is important, to help understand and reduce the disparities in PTB.

The existing literature suggests that disparities in PTB are largely attributable to environmental factors rather than genetic variation (3, 6, 7). These include social stressors, physical stressors (such as environmental chemicals and pollutants), neighborhood variation, healthcare access/quality, and individual cultural practices (8). One study suggested that certain sociodemographic and perinatal health factors contributed to the Black vs. White disparity in PTB, although they reported that more than 60% of the disparities in PTB remained unexplained (9). Other studies also showed that the Black vs. White disparities in PTB persisted after accounting for socioeconomic status, access to care, or medical interventions (10–12). For environmental pollutants, multiple studies revealed associations of air pollution, lead, phthalates, and other chemicals with increased risk of PTB, and found higher exposure levels among non-Hispanic Black women compared with the non-Hispanic White women (13–20). However, it remains unclear whether and what proportion of racial and ethnic disparities in PTB is attributable to different exposures to these environmental factors. A causal mediation analysis (21) is needed to further explore the role of multiple environmental factors on the racial and ethnic disparities in PTB.

There have been recent calls for and developments in the methodology of evaluating environmental factors as potential mediators of health disparities (22–24). Furthermore, given that people are often simultaneously exposed to multiple environmental factors (25, 26), a growing body of conceptual models and statistical methods integrating the joint effects of multiple pollutants into a mediation analysis framework has been proposed, especially in the field of environmental health disparities (27–30). These methods can help quantify the proportion of disparity due to environmental factors, as well as the proportion of disparity that would remain if interventions were made to reduce the levels of these environmental factors. Despite the discussions on this framework and the related methods, a real-world, population-based application of these methods in evaluating the contribution of environmental chemicals/pollutants as a mixture to a health disparity question remains lacking.

**Figure 1:**
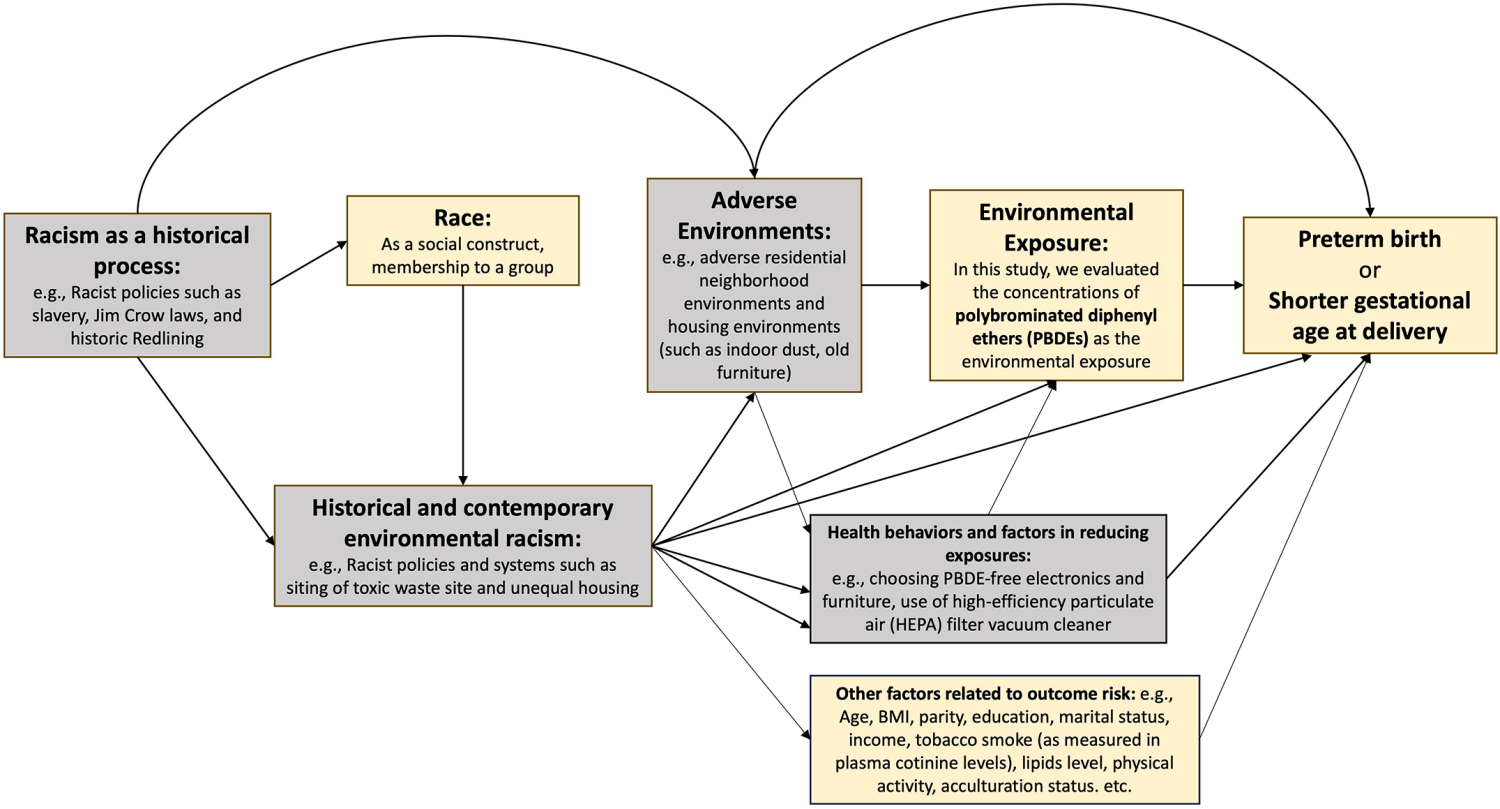
Causal diagram for studying racial and ethnic disparities in preterm birth and gestatonal age at delivery, mediated by polybrominated diphenyl ethers. The boxes in yellow correspond to the variables that we were able to measure with available data in this study. The boxes in gray correspond to the variables on the causal pathway that we were not able to measure in this study.

One class of environmental chemicals, known as polybrominated diphenyl ethers (PBDEs), has been used as a flame retardant since the 1970s and remains to be detected in the US population even a decade after the voluntary phase out that began in 2004 (31–33). PBDEs have the potential to shed or volatilize into the environment (34). Human beings are exposed to PBDEs via inhalation of contaminated air, ingestion of contaminated food, and contact with indoor dust. We hypothesize that PBDEs might be potential mediators for the racial and ethnic disparities in PTB given the following evidence: (1) multiple studies showed higher exposure levels to PBDEs among non-Hispanic Black women compared with non-Hispanic White women (35–37); (2) studies have found associations between certain PBDE congeners and elevated risk of PTB (38–42). In this study, we aimed to use real-world data from a large, multicenter, multiracial/ethnic cohort of singleton pregnancies in the United States to evaluate whether and the extent to which exposure to PBDEs may contribute to the racial and ethnic disparity in PTB and gestational age at delivery, through applying causal mediation analyses incorporating these chemicals (individually and as mixtures) as potential mediators. Race and ethnicity are socially constructed, and racial/ethnic health disparities are driven by the root cause of structural/institutional racism. With that in mind, we present a causal diagram (43) of our research questions in Figure 1.

## 2 Methods

### 2.1 Study population

The study used data from the *Eunice Kennedy Shriver* National Institute of Child Health and Human Development (NICHD) Fetal Growth Studies—Singleton Cohort, a multicenter, multiracial/ethnic prospective study of 2,802 pregnant women recruited during 2009–2013 from 12 US clinical sites (44). Women aged 18–40 years with a singleton pregnancy were enrolled during 8–13 weeks of gestation and followed through delivery. Further details of the cohort can be found elsewhere (44, 45). For this study, we restricted to a subcohort of 2008 eligible women with a low-risk pregnancy (i.e., those with certain pre-existing medical conditions such as systemic diseases or past pregnancy complications were excluded from enrolling in the study) (44, 45) and without obesity [i.e., individuals whose body mass index (BMI) < 30 kg/m^2^], who had available data on gestational age at delivery and measurements of PBDEs from blood specimens. The rationale of these criteria and the numbers excluded are summarized in the Supplementary Material (Supplemental eMethod). Approval for human subjects’ research was obtained from the institutional review boards at all participating sites, and all participants provided informed consent.

### 2.2 Race and ethnicity

Self-identified race and ethnicity were collected at baseline in four categories: non-Hispanic White, non-Hispanic Black, Hispanic, and Asian/Pacific Islander. Further specifications such as self-reported Hispanic origin or Asian background were evaluated in secondary analyses. Non-Hispanic White was defined as the reference group. Too few Hispanic White (*n* = 4) and Hispanic Black (*n* = 4) participants were included to consider these groups separately. We use the self-identified race and ethnicity as the “predictor” parameter in the mediation analysis, while recognizing that race is a social construct (46) that may through racism impact differences in exposures to PBDEs and their sources, as well as differences in factors contributing to PTB or shorter gestational age at delivery, including pathophysiology and access to/quality of prenatal care (7) (Figure 1). As race and ethnicity are non-manipulable, the effect estimates from the mediation analysis should be interpreted as associations reflecting disparity-related (instead of causal/biological) information (23), but we maintained the usage of “effects” when describing these measures to be consistent with common causal mediation terminologies.

### 2.3 Outcomes

The primary outcomes of interest were: (1) gestational age at delivery (weeks), calculated as the difference between date of delivery (abstracted from medical records) and self-reported date of first day of last menstrual period (LMP) as validated by ultrasound (47); and (2) a binary outcome of PTB, defined as delivery prior to 37 weeks of gestation. As secondary outcomes, PTB was further categorized as very early or moderate (<34 weeks) and late (34 to <37 weeks) PTB.

### 2.4 Mediators

A set of potential mediators was determined based on prior knowledge (42), which included plasma concentrations of polybrominated biphenyl (PBB) 153 and 9 PBDEs (PBDE 28, 47, 85, 99, 100, 153, 154, 183, and 209) collected upon enrollment (median: 11 weeks of gestation). Details of the processing, measurement, and limits of quantification (LOQs) of these chemicals have been reported previously (48). All chemical concentrations were reported as ng/mL plasma. For this analysis, we restricted to six PBDEs with quantification rates >30% in this population, including PBDE 28, 47, 99, 100, 153, and 154. Machine-observed values were used for all chemicals in the analysis without substitution, including concentrations below the LOQ (49).

### 2.5 Covariates

The following covariates (collected from the baseline questionnaire unless otherwise specified) were incorporated into our mediation analyses, based on a priori knowledge of being potential confounders for the mediator-outcome associations: maternal age (years); prepregnancy BMI (kg/m^2^), calculated from self-recalled prepregnancy weight divided by measured height squared (50); parity (0, 1, 2+); education level (college degree, some college/undergraduate, graduate/postgraduate); marital status (married or living with partner, not married); family income during last year (<$30,000, $30,000–$49,999, $50,000–$99,999, ≥$100,000, not reported); plasma cotinine level (ng/mL), measured in specimens collected at enrollment (35); plasma total lipids (non-fasting) (ng/mL) at enrollment, quantified using commercially available enzymatic methods (51), and calculated as total cholesterol × 2.27 + triglycerides + 62.3 (52); total and sedentary activities [metabolic equivalent of task (MET) hours/week]; and acculturation status (US-born, recent immigrant, long-term immigrant) based on previous definitions (53). It is possible that race and ethnicity are associated with various downstream risk factors, which might violate the assumption of no mediator-outcome confounders affected by the exposure (54). To address this, we conducted sensitivity analyses using more generalized approaches (23), with details described in the statistical analysis.

### 2.6 Statistical analysis

#### 2.6.1 Descriptive analysis

The characteristics of the study population were summarized with means ± standard deviations or numbers (percentages). Geometric means (GMs) and 95% confidence intervals (CIs) of lipid-adjusted PBDE congener concentrations and their molar sum (∑PBDEs) were calculated, stratified by race and ethnicity and by PTB status.

#### 2.6.2 Mediation analysis

For mediation analysis, we natural log-transformed the machine-observed values of the chemical concentrations to account for skewedness of their distributions, and then performed standardization (subtracted the mean and divided by the standard deviation) to generate comparable scales. The total racial and ethnic (denoted by X) disparity in PTB or gestational age at delivery (denoted by Y) accounting for a set of covariates (denoted by C) was calculated using: $E[Y|X,\;C]=\;\alpha_{0}+\alpha_{1}X+\alpha_{2}C$ (when *Y* represents continuous gestational age at delivery, in weeks), or $logit\{Pr[Y=1|X,\;C]\}=\;\alpha_{0}+\alpha_{1}X+\alpha_{2}C$ (where Y=1 represents PTB and Y=0 represents non-PTB). The following forms of mediator(s) were then evaluated within a counterfactual framework using causal mediation models (for simplicity, we use a continuous variable *Y* as an illustration).

##### 2.6.2.1 Reducing the PBDEs mixtures to a single mediator—weighted quantile sum

As the first approach, we reduced the dimensions of the PBDEs mixtures to a single summary index score via the weighted quantile sum (WQS) approach, which is a method that constructs a weighted index estimating the mixture effect associated with all predictor variables on an outcome (55). The weights for each PBDE were empirically determined using a 40%/60% split of training/validation sets from the data and 500 bootstrap samples for parameter estimation. Next, the WQS index was treated as a single summary measure of the PBDE congeners, and was included as a single mediator in the following models:

$$E[Y|X,\;\mathrm{WQS},C]=\;\alpha_{0}^{\prime }+\alpha_{1}^{\prime }X+\alpha_{2}^{\prime }\mathrm{WQS}+\alpha_{3}^{\prime }C$$

$$E[\mathrm{WQS}|X,\;C]=\beta_{0}^{\prime }+\beta_{1}^{\prime }X+\beta_{2}^{\prime }C$$

The direct and indirect effects through this single mediator were estimated using standard regression-based methods (56).

##### 2.6.2.2 Reducing the number of mediators—select specific mediator(s)

As the second approach, we reduced the number of mediators by selecting a single specific mediator based on the results of a previous study utilizing data from the same cohort of individuals, where multiple statistical approaches [including generalized linear models, principal component analysis, and Bayesian kernel machine regression (BKMR) (57)] have consistently demonstrated PBDE 153 being the main congener associated with shorter gestation and higher risk of PTB, after adjusting for race/ethnicity and other covariates (42). In this study, we further utilized a hierarchical BKMR variable selection approach based on correlation structures of PBDEs in this cohort (which address the potential bias introduced by highly correlated chemicals) to re-evaluate that PBDE 153 is the most important contributor that is associated with gestational age at delivery.

In this approach, we used a single mediator (PBDE 153 as an example) in the following models:

$$E[Y|X,\;\mathrm{PBDE}\;153,C]=\;\alpha_{0}^{*}+\alpha_{1}^{*}X+\alpha_{2}^{*}\mathrm{PBDE}\;153+\alpha_{3}^{*}C$$

$$E[\mathrm{PBDE}\;153|X,\;C]=\beta_{0}^{*}+\beta_{1}^{*}X+\beta_{2}^{*}C$$

The direct and indirect effects through this single mediator were estimated using regression-based methods (56).

##### 2.6.2.3 Modeling all six PBDE congeners as multiple mediators—multiple regression

As the third approach, we included PBDE 28, 47, 99, 100, 153, and 154 simultaneously in the same model:

$$\begin{array}{rl} & E\left[Y|X,\;\mathrm{PBDE}s\;28,\;47,\;99,\;100,\;153,\;154,\;C\right]\\ & \;\;\;\;=\alpha_{0}^{\prime \prime }+\alpha_{1}^{\prime \prime }X+\alpha_{2}^{\prime \prime }\mathrm{PBDE}\;28+\;\alpha_{3}^{\prime \prime }\mathrm{PBDE}\;47\\ & \;\;\;\;+\alpha_{4}^{\prime \prime }\mathrm{PBDE}\;99+\alpha_{5}^{\prime \prime }\mathrm{PBDE}\;100\\ & \;\;\;\;+\alpha_{6}^{\prime \prime }\mathrm{PBDE}\;153+\alpha_{7}^{\prime \prime }\mathrm{PBDE}\;154+\alpha_{8}^{\prime \prime }C \end{array}$$

along with six separate regression models estimating each mediator as a function of the exposure:

$$E[\mathrm{PBDE}\;28|X,\;C]=\beta_{0\_28}^{\prime \prime }+\beta_{1\_28}^{\prime \prime }X+\beta_{2\_28}^{\prime \prime }C$$

$$E[\mathrm{PBDE}\;47|X,\;C]=\beta_{0\_47}^{\prime \prime }+\beta_{1\_47}^{\prime \prime }X+\beta_{2\_47}^{\prime \prime }C$$

$$E[\mathrm{PBDE}\;99|X,\;C]=\beta_{0\_99}^{\prime \prime }+\beta_{1\_99}^{\prime \prime }X+\beta_{2\_99}^{\prime \prime }C$$

$$E[\mathrm{PBDE}\;100|X,\;C]=\beta_{0\_100}^{\prime \prime }+\beta_{1\_100}^{\prime \prime }X+\beta_{2\_100}^{\prime \prime }C$$

$$E[\mathrm{PBDE}\;153|X,\;C]=\beta_{0\_153}^{\prime \prime }+\beta_{1\_153}^{\prime \prime }X+\beta_{2\_153}^{\prime \prime }C$$

$$E[\mathrm{PBDE}\;154|X,\;C]=\beta_{0\_154}^{\prime \prime }+\beta_{1\_154}^{\prime \prime }X+\beta_{2\_154}^{\prime \prime }C$$

The direct and indirect effects (specifically, the joint mediated effect through the set of mediators) were estimated using regression-based methods for multiple mediators (58).

In all the approaches, we estimated the following measures of the disparities in gestational age at delivery and PTB mediated by PBDEs, comparing each of the race and ethnicity groups to the non-Hispanic White group: the total effect (TE), the controlled direct effects (CDEs) while fixing the mediator(s) at various levels, the natural direct and indirect effects (NDE; NIE), and the overall percent mediated (PM) calculated as (NIE/TE) × 100%. All models used regression-based methods, and 95% CIs were obtained via the delta method (from closed-form parameter function estimation in single-mediator models) or bootstrapping (from direct counterfactual imputation estimation in multiple-mediator models). We further extended the models to allow for potential exposure–mediator or mediator–mediator interaction (29, 56, 59).

#### 2.6.3 Secondary and sensitivity analysis

As secondary or sensitivity analyses, we evaluated the outcomes and mediator (WQS index) stratified by finer specifications of race and ethnicity including Hispanic origin or Asian background. We further conducted mediation analysis comparing selective subgroups to non-Hispanic White women. We also performed mediation analysis for PTB subcategories (very early/moderate PTB and late PTB). Furthermore, we evaluated mediation through the WQS index for the absolute risk difference (RD) of PTB using the g-formula approach (60).

Given that some of the proposed mediator-outcome confounders might be downstream factors of racism, hence potentially having an association with race and ethnicity, we conducted sensitivity analyses using the more generalized g-formula approach (23, 60–62), which allowed for a vector of the mediator-outcome confounders potentially affected by the exposure to be accounted for in the analysis.

We also performed the following analyses to evaluate the robustness of our main findings. First, we modeled the WQS index as a binary mediator (≥median vs. <median). Second, we evaluated potential non-linearity via categorizing the PBDEs into <LOQ and quartiles above LOQ, and the WQS index into quintiles, and we used these quantile measures as mediators. Given WQS regression's assumption of unidirectionality, we in addition explored the application of quantile g-computation (63), a flexible extension of WQS estimating the joint effects of a mixture while allowing for chemicals to act on both directions, although with the limitation of being subject to multicollinearity in the presence of highly correlated chemicals within a mixture (64). From the quantile g-computation results, we identified the PBDEs that contributed to the associations with shorter gestational age at delivery, and further created a weighted index of these chemicals as a mediator. Lastly, we conducted sensitivity analysis considering potential measurement errors of the mediator (65).

#### 2.6.4 Statistical software

All causal mediation analyses were conducted using the CMAverse (v.0.1.0) package in R (https://bs1125.github.io/CMAverse/) (62). The WQS analyses were conducted using the gWQS (v.3.0.0) package in R (https://cran.r-project.org/web/packages/gWQS) (66).

## 3 Results

Among the 2008 women included in the study, 552 (27.5%) self-identified as non-Hispanic White, 504 (25.1%) self-identified as non-Hispanic Black, 568 (28.3%) self-identified as Hispanic, and 384 (19.1%) self-identified as Asian/Pacific Islander (Table 1). There were several differences in characteristics across these groups (Table 1). On average, compared with non-Hispanic White women, non-Hispanic Black women were younger, had higher BMI, lower education level, and less family income, and were more likely to be unmarried. Non-Hispanic Black women also had the highest plasma cotinine level and total and sedentary activity levels compared with other groups. Hispanic women had the highest mean BMI and plasma total lipid level, the lowest percentage of being nulliparous, and the highest percentages of attaining less than a college degree or being long-term immigrants. Asian/Pacific Islander women had the highest mean age, the lowest mean BMI, plasma cotinine level, and total activity level, as well as the highest percentage of being recent immigrants. Non-Hispanic Black women had shorter mean gestational ages at delivery (39.0 vs. 39.3 weeks) and higher risks of PTB (9.1% vs. 5.1%) compared with non-Hispanic White women. The outcomes among Hispanic or Asian/Pacific Islander women were similar to those of the non-Hispanic White women.

**Table 1:** Characteristics of the study population by race and ethnicity, NICHD Fetal Growth Study–Singleton Cohort (*n* = 2,008).

	Overall	Non-Hispanic White	Non-Hispanic Black	Hispanic	Asian/Pacific Islander
	(*n* = 2,008)	(*n* = 552)	(*n* = 504)	(*n* = 568)	(*n* = 384)
Age (years)	28.3 ± 5.4	30.3 ± 4.4	25.6 ± 5.5	27.1 ± 5.5	30.6 ± 4.5
Prepregnancy BMI (kg/m^2^)	23.6 ± 3.0	23.3 ± 2.8	24.2 ± 3.1	24.4 ± 2.8	22.2 ± 2.6
Parity, *n* (%)					
0	979 (48.8)	299 (54.2)	253 (50.2)	223 (39.3)	204 (53.1)
1	689 (34.3)	184 (33.3)	157 (31.2)	204 (35.9)	144 (37.5)
2+	340 (16.9)	69 (12.5)	94 (18.7)	141 (24.8)	36 (9.4)
Education level					
Less than college degree	554 (27.6)	30 (5.4)	191 (37.9)	267 (47.0)	66 (17.2)
Some college or undergraduate	1,086 (54.1)	336 (60.9)	271 (53.8)	282 (49.6)	197 (51.3)
Graduate or postgraduate	368 (18.3)	186 (33.7)	42 (8.3)	19 (3.3)	121 (31.5)
Marital status^a^, *n* (%)					
Married or living with partner	1,539 (76.6)	518 (93.8)	251 (49.8)	417 (73.4)	353 (91.9)
Not married	467 (23.3)	33 (6.0)	252 (50.0)	151 (26.6)	31 (8.1)
Family income during last year, *n* (%)					
Less than $30,000	470 (23.4)	21 (3.8)	205 (40.7)	196 (34.5)	48 (12.5)
$30,000–$49,999	288 (14.3)	40 (7.2)	90 (17.9)	125 (22.0)	33 (8.6)
$50,000–$99,999	451 (22.5)	166 (30.1)	86 (17.1)	95 (16.7)	104 (27.1)
$100,000 or more	523 (26.0)	305 (55.3)	57 (11.3)	54 (9.5)	107 (27.9)
Unknown	276 (13.7)	20 (3.6)	66 (13.1)	98 (17.3)	92 (24.0)
Plasma cotinine (ng/mL)^a^	1.1 ± 12.7	1.1 ± 13.2	2.7 ± 20.8	0.3 ± 4.4	0.0 ± 0.2
Plasma total lipids (non-fasting) (mg/dL)^a^^,^^b^	610.5 ± 98.7	613.3 ± 95.9	580.8 ± 99.0	628.3 ± 100.4	619.2 ± 91.2
Total activity (MET hours per week)^a^	323.1 ± 167.8	326.0 ± 147.8	354.8 ± 200.6	307.3 ± 158.9	300.3 ± 153.9
Sedentary activity (MET hours per week)^a^	26.1 ± 18.3	20.7 ± 12.2	37.1 ± 22.2	22.6 ± 17.0	24.4 ± 15.6
Acculturation^a^, *n* (%)					
US-born	1,322 (65.8)	514 (93.1)	462 (91.7)	242 (42.6)	104 (27.1)
Recent immigrant (<10 years)	304 (15.1)	16 (2.9)	16 (3.2)	117 (20.6)	155 (40.4)
Long-term immigrant (≥10 years)	379 (18.9)	20 (3.6)	26 (5.2)	208 (36.6)	125 (32.6)
Gestational age at delivery (weeks)	39.2 ± 1.7	39.3 ± 1.5	39.0 ± 2.1	39.3 ± 1.5	39.3 ± 1.3
Preterm birth, *n* (%)	118 (5.9)	28 (5.1)	46 (9.1)	26 (4.6)	18 (4.7)

Means ± SD for continuous variables. *N* (%) for categorical variables.

^a^ Numbers may not add up to total numbers owing to missing values. Variables with missing values (missing rate) included: marital status (0.1%), plasma cotinine (1.6%), plasma total lipids (1.1%), AHEI 2010 score (37.4%), total activity (0.2%), sedentary activity (0.2%), and acculturation (0.1%).

^b^ Total lipids = total cholesterol × 2.27 + triglycerides + 62.3.

In Table 2, non-Hispanic Black women have higher GMs of all six PBDE congeners and ∑PBDEs than non-Hispanic White women. Across all groups, Hispanic and Asian/Pacific Islander women had the highest GMs of PBDE 28 and PBDE 154, respectively. When comparing those with vs. without PTB in the study population, four PBDEs (i.e., PBDE 28, 47, 100, and 153) and ∑PBDEs had higher GMs.

**Table 2:** Geometric means (95% confidence intervals) of lipid-adjusted PBDE concentrations, stratified by race and ethnicity and by preterm birth status.

Chemicals, ng/g lipid	Race and ethnicity				PTB status	
	Non-Hispanic White	Non-Hispanic Black	Hispanic	Asian/Pacific Islander	Preterm	Non-preterm
	(*n* = 552)	(*n* = 504)	(*n* = 568)	(*n* = 384)	(*n* = 118)	(*n* = 1,890)
PBDE 28	0.20 (0.18, 0.22)	0.29 (0.26, 0.33)	0.30 (0.26, 0.33)	0.25 (0.22, 0.29)	0.28 (0.22, 0.37)	0.25 (0.24, 0.27)
PBDE 47	4.48 (3.77, 5.33)	8.89 (7.49, 10.54)	5.53 (4.55, 6.72)	3.62 (2.88, 4.55)	6.10 (4.12, 9.03)	5.38 (4.87, 5.94)
PBDE 99	0.18 (0.13, 0.25)	0.52 (0.38, 0.73)	0.43 (0.32, 0.58)	0.24 (0.16, 0.34)	0.29 (0.14, 0.58)	0.32 (0.27, 0.38)
PBDE 100	0.55 (0.46, 0.67)	1.44 (1.19, 1.75)	1.01 (0.85, 1.21)	0.52 (0.41, 0.65)	0.84 (0.54, 1.30)	0.82 (0.74, 0.91)
PBDE 153	1.37 (1.16, 1.61)	1.90 (1.61, 2.25)	0.83 (0.73, 0.94)	0.83 (0.71, 0.98)	1.35 (0.95, 1.93)	1.16 (1.07, 1.26)
PBDE 154	0.05 (0.04, 0.07)	0.11 (0.08, 0.15)	0.07 (0.05, 0.09)	0.12 (0.09, 0.17)	0.07 (0.04, 0.14)	0.08 (0.07, 0.09)
∑PBDEs, pmol/g lipid^a^	27.8 (25.2, 30.8)	47.4 (42.8, 52.5)	32.7 (29.6, 36.1)	23.4 (20.6, 26.5)	36.6 (29.4, 45.6)	31.9 (30.2, 33.7)

Geometric means (95% CI) calculated using all machine-observed values including those below the LOQs, where zero and negative values were assigned the value of (lowest positive value)/2. PTB, preterm birth.

^a^ ∑PBDEs (in pmol/g lipid) refers to the molar sum of PBDEs, which was calculated by dividing each lipid-adjusted chemical concentration by its molecular weight and summing all detectable concentrations.

**Figure 2:**
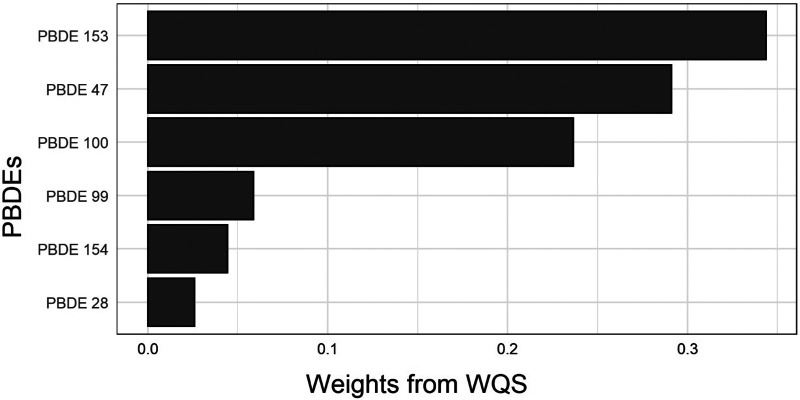
Weights for each PBDE congener from the weighted quantile sum index. WQS, weighted quantile sum.

We used a WQS index to estimate the mixture effect of six PBDEs on gestational age at delivery (weights for each PBDE shown in Figure 2). The association of a 1-unit increase in the WQS index with gestational age at delivery was *β* (95% CI) = −0.20 (−0.35, −0.05) weeks, adjusted for race and ethnicity and other covariates. Table 3 provides results from the mediation analysis, where the WQS index was considered a potential mediator for the racial and ethnic disparity of gestational age at delivery or PTB. Comparing non-Hispanic Black women with non-Hispanic White women, the covariate-adjusted difference in gestational age at delivery was *β*_TE_ (95% CI) = −0.30 (−0.54, −0.05) weeks. The CDEs (95% CIs) when fixing everyone's WQS index levels at the 25th, 50th, and 75th percentiles were −0.18 (−0.46, 0.10), −0.23 (−0.49, 0.02), and −0.32 (−0.57, −0.07) weeks, respectively. Overall, a suggestive NIE of *β*_NIE_ (95% CI) = −0.04 (−0.07, 0.00) weeks were mediated through the WQS index (proportion mediated = 11.8%). The odds ratio of PTB comparing non-Hispanic Black with non-Hispanic White women was OR_TE_ (95% CI) = 1.82 (1.00, 3.31), yet no statistically significant NIE was found. In addition, no statistically significant disparity was found when comparing Hispanic or Asian/Pacific Islander women with non-Hispanic White women.

**Table 3:** Estimates of direct and indirect effects mediated through a weighted quantile sum exposure index of PBDEs for the associations of race and ethnicity with gestational age at delivery and preterm birth.

Race and ethnicity	Adjusted^a^ β (95% CI^b^) for gestational age at delivery, weeks						
	Natural direct effect (*β*_NDE_)	Natural indirect effect (*β*_NIE_)	Controlled direct effects (CDEs), fixing the WQS index at the 25th percentile, median, and 75th percentile			Total effect(*β*_TE_)	Proportion mediated (PM), %
			*β*_CDE(25th)_	*β*_CDE(median)_	*β*_CDE(75th)_		
Non-Hispanic White	REF	REF	REF	REF	REF	REF	REF
Non-Hispanic Black	−0.26 (−0.51, −0.01)	−0.04 (−0.07, 0.00)	−0.18 (−0.46, 0.10)	−0.23 (−0.49, 0.02)	−0.32 (−0.57, −0.07)	−0.30 (−0.54, −0.05)	11.8% (−3.8%, 27.5%)
Hispanic	0.07 (−0.18, 0.32)	−0.01 (−0.03, 0.01)	0.12 (−0.16, 0.40)	0.09 (−0.17, 0.34)	0.03 (−0.23, 0.29)	0.06 (−0.19, 0.31)	−20.7% (−123.7%, 82.2%)
Asian/Pacific Islander	−0.05 (−0.32, 0.21)	−0.01 (−0.03, 0.02)	−0.02 (−0.32, 0.28)	−0.04 (−0.31, 0.23)	−0.08 (−0.35, 0.20)	−0.06 (−0.32, 0.20)	13.1% (−50.6%, 76.7%)
	Adjusted^a^ OR (95% CI^b^) for preterm birth						
	Natural direct effect (OR_NDE_)	Natural indirect effect (OR_NIE_)	Controlled direct effects (CDEs), fixing the WQS index at the 25th percentile, median, and 75th percentile			Total effect (OR_TE_)	Proportion mediated (PM), %
			OR_CDE(25th)_	OR_CDE(median)_	OR_CDE(75th)_		
Non-Hispanic White	REF	REF	REF	REF	REF	REF	REF
Non-Hispanic Black	1.79 (0.98, 3.25)	1.02 (0.98, 1.06)	1.77 (0.89, 3.52)	1.78 (0.95, 3.32)	1.79 (0.98, 3.25)	1.82 (1.00, 3.31)	4.0% (−5.1%, 13.1%)
Hispanic	0.89 (0.45, 1.75)	1.01 (0.99, 1.03)	0.83 (0.38, 1.83)	0.85 (0.42, 1.73)	0.89 (0.45, 1.75)	0.90 (0.46, 1.77)	−7.4% (−60.1%, 45.2%)
Asian/Pacific Islander	0.97 (0.47, 2.01)	1.01 (0.98, 1.03)	0.81 (0.35, 1.87)	0.88 (0.41, 1.87)	0.96 (0.47, 1.99)	0.97 (0.47, 2.01)	−29.6% (−807.4%, 748.3%)

^a^ Adjusted for maternal age (years), prepregnancy BMI (kg/m^2^), parity (0, 1, 2+), education level (<college degree, some college/undergraduate, graduate/postgraduate), marital status (married or living with partner, not married), family income during last year (<$30,000, $30,000–$49,999, $50,000–$99,999, $100,000 or more, not reported), plasma cotinine level (ng/mL), plasma total lipids (ng/mL), total activity (MET hours per week), sedentary activity (MET hours per week), and acculturation (US born, recent immigrant, long-term immigrant). Observations with missing covariates were excluded from the adjusted models.

^b^ Standard errors for calculating the 95% CIs obtained using the delta method, based on point estimates obtained using closed-form parameter function estimation.

The correlation coefficients between PBDEs are shown in Supplementary Figure S1. PBDE 28, 47, 99, and 100 were moderately to highly correlated, and PBDE 153 and 154 were weakly correlated. Using BKMR with hierarchical variable selection (based on the correlation structure, PBDE 28, 47, 99, and 100 were assigned as Group 1, and PBDE 153 and 154 were assigned as Group 2), we found that Group 2 was of relatively greater importance, and PBDE 153 was the most important chemical within Group 2 [reflected by the posterior inclusion probabilities (PIPs) shown in Supplementary Table S1] that was associated with shorter gestational age at delivery (Supplementary Figure S2). There were no qualitative interactions between the PBDEs (Supplementary Figure S3). Thus, for the single-mediator model, we included PBDE 153 as the mediator. Table 4 provides results from the mediation analysis, where only PBDE 153 was considered as a potential mediator. The CDEs (95% CIs) when fixing everyone's PBDE 153 levels at the 25th, 50th, and 75th percentiles were −0.18 (−0.45, 0.09), −0.18 (−0.45, 0.09), and −0.29 (−0.54, −0.05) weeks, respectively, yet with a non-significant NIE mediated via PBDE 153 (proportion mediated = 7.9%). No statistically significant NIE was found for the non-Hispanic Black vs. non-Hispanic White disparity in PTB.

**Table 4:** Estimates of direct and indirect effects mediated through PBDE 153 concentrations for the associations of race and ethnicity with gestational age at delivery and preterm birth.

Race and ethnicity	Adjusted^a^ β (95% CI^b^) for gestational age at delivery, weeks						
	Natural direct effect (*β*_NDE_)	Natural indirect effect (*β*_NIE_)	Controlled direct effects (CDEs), fixing PBDE 153 concentration at the 25th percentile, median, and 75th percentile			Total effect (*β*_TE_)	Proportion mediated (PM), %
			*β*_CDE(25th)_	*β*_CDE(median)_	*β*_CDE(75th)_		
Non-Hispanic White	REF	REF	REF	REF	REF	REF	REF
Non-Hispanic Black	−0.27 (−0.52, −0.03)	−0.02 (−0.06, 0.01)	−0.18 (−0.45, 0.09)	−0.18 (−0.45, 0.09)	−0.29 (−0.54, −0.05)	−0.30 (−0.54, −0.05)	7.9% (−5.4%, 21.1%)
Hispanic	0.05 (−0.20, 0.30)	0.01 (−0.01, 0.03)	0.07 (−0.21, 0.34)	0.07 (−0.21, 0.34)	0.05 (−0.21, 0.30)	0.06 (−0.19, 0.31)	18.4% (−52.2%, 77.6%)
Asian/Pacific Islander	−0.07 (−0.33, 0.19)	0.01 (−0.01, 0.02)	−0.06 (−0.35, 0.23)	−0.06 (−0.35, 0.23)	−0.07 (−0.33, 0.19)	−0.06 (−0.32, 0.20)	−9.0% (−57.8%, 39.8%)
	Adjusted^a^ OR (95% CI^b^) for preterm birth (cutoff: 37 weeks)						
	Natural direct effect (OR_NDE_)	Natural indirect effect (OR_NIE_)	Controlled direct effects (CDEs), fixing PBDE 153 concentration at the 25th percentile, median, and 75th percentile			Total effect (OR_TE_)	Proportion mediated (PM), %
			OR_CDE(25th)_	OR_CDE(median)_	OR_CDE(75th)_		
Non-Hispanic White	REF	REF	REF	REF	REF	REF	REF
Non-Hispanic Black	1.81 (0.99, 3.32)	1.01 (0.98, 1.04)	1.92 (0.97, 3.79)	1.92 (0.97, 3.79)	1.81 (0.99, 3.31)	1.82 (1.00, 3.33)	1.3% (−5.0%, 7.6%)
Hispanic	0.89 (0.45, 1.78)	0.99 (0.94, 1.04)	0.91 (0.42, 1.97)	0.91 (0.42, 1.97)	0.89 (0.45, 1.78)	0.88 (0.45, 1.76)	7.3% (−50.4%, 64.9%)
Asian/Pacific Islander	0.98 (0.48, 2.03)	0.99 (0.97, 1.01)	0.90 (0.39, 2.07)	0.90 (0.39, 2.07)	0.98 (0.48, 2.03)	0.97 (0.47, 2.01)	33.9% (−911.4%, 979.2%)

^a^ Adjusted for maternal age (years), prepregnancy BMI (kg/m^2^), parity (0, 1, 2+), education level (<college degree, some college/undergraduate, graduate/postgraduate), marital status (married or living with partner, not married), family income during last year (<$30,000, $30,000–$49,999, $50,000–$99,999, $100,000, or more, not reported), plasma cotinine level (ng/mL), plasma total lipids (ng/mL), total activity (MET hours per week), sedentary activity (MET hours per week), and acculturation (US born, recent immigrant, long-term immigrant). Observations with missing covariates were excluded from the adjusted models.

^b^ Standard errors for calculating the 95% CIs obtained using the delta method, based on point estimates obtained using closed-form parameter function estimation.

Table 5 provides results when all six PBDEs were included simultaneously as multiple mediators. The CDEs (95% CIs) when fixing all PBDEs at the 25th, 50th, and 75th percentiles were −0.25 (−0.53, 0.02), −0.14 (−0.47, 0.17), and −0.27 (−0.61, −0.03) weeks, respectively, yet with a non-significant NIE jointly mediated via PBDE 28, 46, 99, 100, 153, and 154 (proportion mediated = 16.3%). No statistically significant NIE was found for the disparity in PTB. No exposure–mediator(s) or mediator–mediator interaction was found for any of the aforementioned analyses (*p*-values for interactions >0.05).

**Table 5:** Estimates of direct and indirect effects mediated through concentrations of all six PBDE congeners (PBDE 28, 47, 99, 100, 153, and 154) for the associations of race and ethnicity with gestational age at delivery and preterm birth.

Race and ethnicity	Adjusted^a^ β (95% CI^b^) for gestational age at delivery, weeks						
	Natural direct effect (*β*_NDE_)	Natural indirect effect (*β*_NIE_)	Controlled direct effects (CDEs), fixing all chemical concentrations at the 25th percentile, median, and 75th percentile			Total effect^c^ (β_TE_)	Proportion mediated (PM), %
			*β*_CDE(25th)_	*β*_CDE(median)_	*β*_CDE(75th)_		
Non-Hispanic White	REF	REF	REF	REF	REF	REF	REF
Non-Hispanic Black	−0.25 (−0.54, −0.02)	−0.05 (−0.16, 0.05)	−0.25 (−0.53, 0.02)	−0.14 (−0.47, 0.17)	−0.27 (−0.61, −0.03)	−0.29 (−0.57, −0.11)	16.3% (−16.2%, 81.1%)
Hispanic	0.05 (−0.19, 0.27)	0.01 (−0.05, 0.08)	0.20 (−0.07, 0.46)	0.23 (−0.02, 0.52)	−0.05 (−0.33, 0.21)	0.06 (−0.17, 0.26)	10.4% (−233.6%, 335.5%)
Asian/Pacific Islander	−0.04 (−0.29, 0.20)	−0.03 (−0.14, 0.05)	0.06 (−0.28, 0.36)	0.12 (−0.22, 0.43)	−0.13 (−0.43, 0.15)	−0.07 (−0.39, 0.18)	44.8% (−203.2%, 212.7%)
	Adjusted^a^ OR (95% CI^b^) for preterm birth						
	Natural direct effect (OR_NDE_)	Natural indirect effect (OR_NIE_)	Controlled direct effects (CDEs), Fixing all chemical concentrations at the 25th percentile, median, and 75th percentile			Total effect^c^ (OR_TE_)	Proportion mediated (PM), %
			OR_CDE(25th)_	OR_CDE(median)_	OR_CDE(75th)_		
Non-Hispanic White	REF	REF	REF	REF	REF	REF	REF
Non-Hispanic Black	1.66 (0.93, 3.16)	1.04 (0.93, 1.15)	1.66 (0.93, 3.21)	1.66 (0.93, 3.20)	1.67 (0.93, 3.19)	1.73 (1.03, 3.04)	9.7% (−27.8%, 47.3%)
Hispanic	0.89 (0.51, 1.75)	1.01 (0.52, 1.74)	0.89 (0.51, 1.76)	0.89 (0.51, 1.76)	0.89 (0.51, 1.75)	0.90 (0.52, 1.74)	−8.7% (−150.4%, 170.8%)
Asian/Pacific Islander	0.97 (0.41, 1.93)	1.00 (0.92, 1.09)	0.97 (0.41, 1.95)	0.97 (0.41, 1.95)	0.97 (0.40, 1.94)	0.97 (0.41, 2.02)	4.8% (−100.2%, 103.3%)

^a^ Adjusted for maternal age (years), pre-pregnancy BMI (kg/m^2^), parity (0, 1, 2+), education level (<college degree, some college/undergraduate, graduate/postgraduate), marital status (married or living with partner, not married), family income during last year (<$30,000, $30,000–$49,999, $50,000–$99,999, $100,000, or more, not reported), plasma cotinine level (ng/ml), plasma total lipids (ng/mL), total activity (MET hours per week), sedentary activity (MET hours per week), and acculturation (US born, recent immigrant, long-term immigrant). Observations with missing covariates were excluded from the adjusted models.

^b^ 95% CIs obtained using the bootstrapping method, based on point estimates obtained using direct counterfactual imputation estimation.

^c^ TE estimates slightly differ from Tables 3, 4 since causal imputation and bootstrap methods were used for models with multiple mediators.

As secondary analysis, the outcomes stratified by further specified Hispanic origin or Asian background are provided in Supplementary Table S2. Several subgroups had shorter mean gestational ages at delivery than non-Hispanic White participants. Among them, those reporting Filipino background also had higher mean WQS index levels than non-Hispanic White women (Supplementary Figure S4). Mediation analysis results comparing Filipino to non-Hispanic White women are provided in Supplementary Table S3. The proportion mediated by WQS index for the shorter gestational age at delivery was 16% (95% CI: −11%, 61%) when comparing the *n* = 45 women with Filipino background with non-Hispanic White women.

The results from the sensitivity analyses to evaluate the robustness of our main results are shown in the Supplementary Materials. In summary, evaluating PTB in subcategories showed no significant findings (Supplementary Table S4), and evaluating the absolute risk of PTB on the risk difference scale showed similar findings (Supplementary Table S5). Using g-estimation yielded similar patterns of mediation (Supplementary Table S6). Modeling the WQS index as a binary mediator dichotomized at the median had very little impact on the indirect effect estimates (Supplementary Table S7). The associations between PBDE 153 or the WQS index and gestational age at delivery were linear (*p*-trend < 0.05, Supplementary Table S8), and modeling WQS or PBDE 153 as quantiles showed similar mediation effects (Supplementary Tables S9, S10). Using a weighted exposure index of 4 PBDEs (PBDE 28,99, 100, and 153) based on quantile g-computation analysis (selecting the PBDEs with weights toward an association with shortened gestational age at delivery, Supplementary Figure S5) yielded similar mediation patterns as the WQS index (Supplementary Table S11). Finally, Supplementary Figure S6 showed similar estimates across various magnitudes of potential mediator measurement error.

## 4 Discussion

In this multiracial/ethnic cohort of pregnant women in the United States, we demonstrated shorter gestational age at delivery, higher risk of PTB, and higher exposure levels to PBDEs among non-Hispanic Black than in non-Hispanic White women, and we evaluated potential mediation by PBDEs for the racial and ethnic disparities in gestational age at delivery and PTB utilizing several recently developed causal mediation approaches. In particular, we observed that a weighted index summarizing PBDEs as a mixture had a suggestive mediating role in the Black vs. White disparity in gestational age at delivery that accounted for 11.8% of the total disparity. No significant mediation was found for the disparity of PTB, or from evaluating other forms of PBDE mediators. We also revealed disparities in gestational age at delivery comparing the Filipino subgroup with non-Hispanic White women, although no significant mediation via PBDEs was found. While further validation using larger datasets are needed, our results point to the possibility of PBDE mixtures acting as mediators for the existing racial and ethnic disparities in gestational age at delivery.

Our observation of a higher risk of PTB and shorter mean gestational age at delivery among non-Hispanic Black women compared with non-Hispanic White women is consistent with previous reports (4, 9, 67). However, the PTB risks were lower than the general population since this study consisted of relatively healthy, non-obese individuals. We also observed higher average concentrations of PBDEs comparing non-Hispanic Black with non-Hispanic White women, which aligned with previous studies that reported similar disparity patterns among certain PBDEs (e.g., 28, 47, 99, and 100) (36, 68). This disparity may be explained by differences in social and contextual factors contributing to sources of PBDE exposures, such as differences in residential neighborhoods (69), housing (e.g., indoor dust) (70), and furniture PBDE exposures (68). Furthermore, we observed higher mean levels of four PBDE congeners and ∑PBDEs in those who delivered preterm compared with non-preterm, suggesting that PBDEs, either individually or as mixtures, might be potential mediator(s) accounting for part of the disparities in PTB or gestational age at delivery. In our causal mediation analyses, we found that a weighted index of all PBDEs (i.e., the WQS index) accounted for 11.8% of the total Black vs. White disparity in gestational age at delivery. Particularly, we found that the CDEs were closer to the null when fixing everyone's WQS index at lower levels, suggesting the potential benefit in reducing the existing disparity in gestational age at delivery by intervening on PBDE levels in the entire population. Conversely, the proportion mediated by the WQS index for the Black vs. White disparity in PTB was only 4% (and non-significant), which could be explained either by lower statistical power owing to a limited number of events, or that the magnitude of mediation for gestational age at delivery might be relatively small to make a noticeable impact on the risk of PTB in this healthier population. Given this is the first study that evaluated the potential mediation role of PBDEs for this disparity, future studies of larger sample sizes or conducted among a higher-risk population might be needed to validate our findings. Past studies have revealed other mediators (such as socioeconomic and health factors, and access to healthcare) for the racial and ethnic disparity in PTB, but a large proportion of the disparity remained (9–11, 71–73). If PBDEs truly mediate part of the racial and ethnic disparity in length of gestation or PTB, then this class of chemicals might be an additional modifiable factor to help further alleviate this disparity.

Similarly to previous literature (5), we did not observe significant differences in gestational age at delivery or PTB comparing Hispanic or Asian/Pacific Islander women with non-Hispanic women. However, we did find a 38 per 1,000 births higher risk of PTB and 0.5-week shorter mean gestational age at delivery comparing a subgroup of Filipino women with non-Hispanic White women, which was consistent with previous studies showing that Filipino women had higher relative risk of PTB (compared with non-Hispanic White) than other Asian subgroups (74). Despite these Filipino women also having higher exposure levels to PBDEs, the results from mediation analysis were non-significant. This might be due to the small number of participants with various Asian backgrounds, although we could not rule out the possibility that there might be unmeasured confounding such as cultural, psychosocial, or early life factors that are driving this disparity, especially when more than half of the Filipino women in this study were immigrants. Future studies with more specific focus on these racial/ethnic minority subgroups that collect acculturation-related variables are needed to further explore this mediation.

In this study, we compared three different approaches of incorporating PBDEs as potential mediators of racial and ethnic disparities in gestational age at delivery and PTB: reducing to a WQS index, selecting a single PBDE 153 congener according to prior knowledge and its relative importance from the hierarchical BKMR selection, and including six PBDE congeners as multiple mediators. Overall, the estimated proportion mediated via the single PBDE 153 congener was smaller than that via the WQS index. This is possibly owing to the limitation of selecting mediator(s) a priori based on the mediator-outcome association alone, which might leave out important mediator(s) weakly associated with the outcome that may also contribute to the indirect effect. The estimated proportion jointly mediated by multiple PBDEs was higher than the proportion mediated by the WQS index, but with much wider CIs due to potential overfitting or multicollinearity. Our example showed that the WQS approach carries the advantage of reducing the PBDEs to a single score to avoid overfitting or multicollinearity, while preserving the information from each congener, serving as a suitable approach to explore the overall contribution of a chemical mixture to a health disparity question (28).

We acknowledge several limitations of this study. First, this study consisted of women with low-risk of adverse health outcomes at baseline and without obesity, so our findings might not be fully generalizable to the overall US population. Second, unmeasured confounding was inevitable, such as other geographic, psychosocial, or lifestyle factors. Third, statistical power was limited when evaluating potential mediation within certain subgroups. Lastly, we were not able to directly measure historical or contemporary environmental racism or adverse environments in these data that are contributing to (or on the causal pathway for) the observed disparities where race and ethnicity act as a proxy for these complex processes (75). Further studies are needed to inform interventions on the policies and systems level.

This study has many unique strengths. First, this study utilized a prospective cohort design in a large, racially/ethnically diverse population with clinically validated outcomes and a comprehensive set of covariates. Second, we applied different statistical approaches to evaluate mediation(s) through individual as well as mixtures of PBDEs. Third, efforts were made to evaluate mediation for disparities in subcategories of PTB, or among other under-studied racial and ethnic subgroups (e.g., based on Asian backgrounds). Lastly, we conducted various sensitivity analyses to validate the robustness of our findings.

## 5 Conclusions

In conclusion, in this multiracial/ethnic cohort of pregnant women in the United States, we found that non-Hispanic Black women had shorter gestational ages at delivery, higher risk of PTB, and higher exposures to PBDEs compared with non-Hispanic White women. Our mediation analysis provided suggestive evidence that the Black vs. White disparity in gestational age at delivery might be partially mediated by disparities in exposures to PBDEs. Lowering the PBDE exposures at the population level may help reduce this disparity.

## Data Availability

The datasets presented in this article are not readily available because of data usage and confidentiality agreements. Requests to access the data should be directed to the corresponding author, the senior author, and the Eunice Kennedy Shriver National Institute of Child Health and Human Development Intramural Research Program. Requests to access the datasets should be directed to Tamarra James-Todd, tjtodd@hsph.harvard.edu.
