# Supplementary material for: Racial and ethnic disparities in preterm birth: a mediation analysis incorporating mixtures of polybrominated diphenyl ethers
Source: Front Reprod Health. 2024 Jan 8;5:1285444. doi: 10.3389/frph.2023.1285444 (PMC10800537; doi:10.3389/frph.2023.1285444)
Supplement: Supplementary file 1 [file Datasheet1.docx]

Supplementary Materials

Racial and ethnic disparities in preterm birth: a mediation analysis incorporating mixtures of polybrominated diphenyl ethers

**Zifan Wang^1*^, Cuilin Zhang^2,3^, Paige L. Williams^4,5^, Andrea Bellavia^1^, Blair J. Wylie^6^, Kurunthachalam Kannan^7^, Michael S. Bloom^8^, Kelly J. Hunt^9^, and Tamarra James-Todd^1,5^**

^1^ Department of Environmental Health, Harvard T.H. Chan School of Public Health, Boston, MA, USA

^2^ Global Center for Asian Women’s Health, Bia-Echo Asia Centre for Reproductive Longevity & Equality (ACRLE), NUS Yong Loo Lin School of Medicine, National University of Singapore, Singapore

^3^ Department of Obstetrics & Gynecology, Yong Loo Lin School of Medicine, National University of Singapore, Singapore

^4^ Department of Biostatistics, Harvard T.H. Chan School of Public Health, Boston, MA, USA

^5^ Department of Epidemiology, Harvard T.H. Chan School of Public Health, Boston, MA, USA

^6^ Department of Obstetrics and Gynecology, Columbia University Vagelos College of Physicians and Surgeons, New York, NY, USA

^7^ Wadsworth Center, New York State Department of Health, Albany, NY, USA

^8^ Department of Global and Community Health, George Mason University, Fairfax, VA, USA

^9^ Department of Public Health Sciences, Medical University of South Carolina, Charleston, SC, USA

# Supplemental Method (Page 2)

# Supplemental tables (Pages 3-15)

# Supplemental figures (Pages 16-21)

Supplemental Method. Further details on the inclusion/exclusion criteria of this study.

For this study, we first restricted to a sub-cohort of 2334 women with a low-risk pregnancy and without obesity across four self-reported race and ethnicity groups (non-Hispanic White, non-Hispanic Black, Hispanic, or Asian/Pacific Islander). The reasons that we excluded the remaining sub-cohort of 468 women with a low-risk pregnancy and with obesity (i.e. pre-pregnancy BMI ≥ 30 kg/m^2^) from this analysis are as follows: (1) the sub-cohort with obesity had different eligibility/exclusion criteria from the sub-cohort without obesity, which may reflect different demographic, lifestyle, and health conditions^1^; (2) the sub-cohort with obesity recruited participants unselected by race/ethnicity^1^, and had a small sample size with limited statistical power for a mediation analysis on racial/ethnic disparities; (3) a prior study using data from this cohort only found associations between PBDEs and gestational age at delivery/PTB among the sub-cohort without obesity^2^.

We then further restricted to 2233 women who remained eligible after enrollment and provided first trimester blood samples with available measurement of PBDEs. We then excluded those without data on gestational age at delivery (n = 182) or had a non-live birth (n = 5). All remaining participants had available information on self-identified race/ethnicity. Lastly, we excluded observations with PBDEs concentrations greater than 5 standard deviations from the mean values to avoid potential distortion from extreme values (n = 38). The final data for analysis consisted of 2008 participants, as described in the manuscript.

**References:**

1. Grewal J, Grantz KL, Zhang C, et al. Cohort Profile: NICHD Fetal Growth Studies–Singletons and Twins. *Int J Epidemiol*. 2018;47(1):25-25l. doi:10.1093/ije/dyx161

2. Wang Z, Zhang C, Williams PL, et al. Polybrominated diphenyl ethers in early pregnancy and preterm birth: Findings from the NICHD Fetal Growth Studies. *Int J Hyg Environ Health*. 2022;243:113978. doi:10.1016/j.ijheh.2022.113978

# **Supplemental Table S1.** Group and conditional posterior inclusion probabilities in the Bayesian kernel machine regression model with hierarchical variable selection.

| **PBDEs** | **Group** | **Group PIP** | **Conditional PIP** |
| --- | --- | --- | --- |
| PBDE 28 | 1 | 0.36 | 0.28 |
| PBDE 47 | 1 | 0.36 | 0.16 |
| PBDE 99 | 1 | 0.36 | 0.22 |
| PBDE 100 | 1 | 0.36 | 0.34 |
| PBDE 153 | 2 | 0.95 | 0.87 |
| PBDE 154 | 2 | 0.95 | 0.13 |

PIP: posterior inclusion probability.

# **Supplemental Table S2.** Outcomes stratified by race and ethnicity, including Hispanic origin and Asian background.

| **Race and ethnicity** | **N** | **Gestational age at delivery (weeks), mean ± SD** | **PTB, n (%)** |
| --- | --- | --- | --- |
| **Non-Hispanic White** | 552 | 39.3 ± 1.5 | 28 (5.1%) |
| **Non-Hispanic Black** | 504 | 39.0 ± 2.1 | 46 (9.1%) |
| **Hispanic, *total*** | 568 | 39.3 ± 1.5 | 26 (4.6%) |
| **Hispanic, *by self-reported Hispanic origin:*** |  |  |  |
| Puerto Rican | 73 | 39.3 ± 1.2 | 3 (4.1%) |
| Cuban/Cuban American | 3 | 39.4 ± 1.5 | 0 (0%) |
| Dominican (Republic) | 94 | 39.4 ± 1.9 | 4 (4.3%) |
| Mexican, Mexican American, Chicano | 265 | 39.3 ± 1.6 | 15 (5.7%) |
| Central or South American | 96 | 39.5 ± 1.2 | 3 (3.7%) |
| Other | 37 | 39.4 ± 1.1 | 1 (2.7%) |
| **Asian/Pacific Islander, *total*** | 384 | 39.3 ± 1.3 | 18 (4.7%) |
| **Asian/Pacific Islander, *by self-reported Asian background ^a^:*** |  |  |  |
| Chinese | 120 | 39.4 ± 1.3 | 3 (2.5%) |
| Filipino | 45 | 38.8 ± 1.3 | 4 (8.9%) |
| Indian Subcontinent (India, Pakistan, Sri Lanka) | 56 | 39.3 ± 1.3 | 3 (5.4%) |
| Japanese | 9 | 39.9 ± 0.7 | 0 (0%) |
| Korean | 49 | 39.7 ± 1.4 | 2 (4.1%) |
| Malay | 2 | 38.7 ± 1.0 | 0 (0%) |
| Thai | 5 | 39.7 ± 0.9 | 0 (0%) |
| Vietnamese | 40 | 39.2 ± 1.2 | 3 (7.5%) |
| Other | 50 | 38.9 ± 1.4 | 3 (6.0%) |

# **Supplemental Table S3.** Estimates of direct and indirect effects mediated through a WQS chemical exposure index of PBDEs for the associations of race and ethnicity with gestational age at delivery and preterm birth, comparing women with Filipino Asian background to non-Hispanic White women.

| **Race and ethnicity** | **Adjusted^a^ β (95% CI^b^) for gestational age at delivery, weeks** | | | | | | |
| --- | --- | --- | --- | --- | --- | --- | --- |
|  | **Natural Direct Effect (β_NDE_)** | **Natural Indirect Effect (β_NIE_)** | **Controlled Direct Effects (CDEs),**  **fixing the WQS index at the 25^th^ percentile, median, and 75^th^ percentile** | | | **Total Effect**  **(β_TE_)** | **Proportion Mediated**  **(PM), %** |
|  |  |  | **β_CDE(25th)_** | **β_CDE(median)_** | **β_CDE(75th)_** |  |  |
| Non-Hispanic White (n = 552) | *REF* | *REF* | *REF* | *REF* | *REF* | *REF* | *REF* |
| Asian/Pacific Islander: Filipino background (n = 45) | -0.42  (-0.85, -0.04) | -0.08  (-0.23, 0.02) | -0.28  (-0.76, 0.10) | -0.37  (-0.80, 0.02) | -0.52  (-0.90, -0.10) | -0.50  (-0.90, -0.11) | 15.5%  (-10.9%, 60.6%) |
|  | **Adjusted^a^ OR (95% CI^b^) for preterm birth** | | | | | | |
|  | **Natural Direct Effect (OR_NDE_)** | **Natural Indirect Effect (OR_NIE_)** | **Controlled Direct Effects (CDEs),**  **Fixing the WQS index at the 25^th^ percentile, median, and 75^th^ percentile** | | | **Total Effect**  **(OR_TE_)** | **Proportion Mediated**  **(PM), %** |
|  |  |  | **OR_CDE(25th)_** | **OR_CDE(median)_** | **OR_CDE(75th)_** |  |  |
| Non-Hispanic White (n = 552) | *REF* | *REF* | *REF* | *REF* | *REF* | *REF* | *REF* |
| Asian/Pacific Islander: Filipino background (n = 45) | 1.59  (0.34, 5.83) | 1.09  (0.67, 1.48) | 1.30  (0.00, 4.44) | 1.41  (0.00, 3.67) | 1.68  (0.00, 4.14) | 1.74  (0.34, 5.47) | 20.2%  (-317.1%, 152.1%) |

^a^ Adjusted for maternal age (years), pre-pregnancy BMI (kg/m^2^), parity (0, 1, 2+), education level (<college degree, some college/undergraduate, graduate/post-graduate), marital status (married or living with partner, not married), family income during last year (<$30,000, $30,000-$49,999, $50,000-$99,999, $100,000 or more, not reported), plasma cotinine level (ng/mL), plasma total lipids (ng/mL), total activity (MET hours per week), sedentary activity (MET hours per week), and acculturation (US-born, recent immigrant, long-term immigrant). Observations with missing covariates were excluded from the adjusted models.

^b^ Standard errors for calculating the 95% CIs obtained using the more robust bootstrap method given small sample sizes.

# **Supplemental Table S4.** Estimates of natural direct and indirect effects mediated through a WQS chemical exposure index of PBDEs for the associations of race and ethnicity with preterm birth sub-categories.

| **Race and ethnicity** | **Adjusted^a^ OR (95% CI^b^)**  **for very early/moderate PTB** (n = 23) | | | |  | **Adjusted^a^ OR (95% CI^b^)**  **for late PTB** (n = 95) | | | |
| --- | --- | --- | --- | --- | --- | --- | --- | --- | --- |
|  | **Natural Direct Effect (OR_NDE_)** | **Natural Indirect Effect (OR_NIE_)** | **Total Effect**  **(OR_TE_)** | **Proportion Mediated**  **(PM), %** |  | **Natural Direct Effect (OR_NDE_)** | **Natural Indirect Effect (OR_NIE_)** | **Total Effect**  **(OR_TE_)** | **Proportion Mediated**  **(PM), %** |
| Non-Hispanic White | *REF* | *REF* | *REF* | *REF* |  | *REF* | *REF* | *REF* | *REF* |
| Non-Hispanic Black | 2.09  (0.53, 8.30) | 1.08  (0.98, 1.19) | 2.25  (0.57, 8.93) | 13.0%  (-8.3%, 34.3%) |  | 1.68  (0.87, 3.25) | 1.00  (0.97, 1.05) | 1.68  (0.87, 3.27) | 1.0%  (-8.7%, 10.7%) |
| Hispanic | 0.42  (0.07, 2.37) | 1.03  (0.98, 1.10) | 0.43  (0.08, 2.45) | -2.5%  (-11.4%, 6.4%) |  | 1.05  (0.51, 2.18) | 1.00  (0.98, 1.02) | 1.05  (0.51, 2.18) | 4.0%  (-62.2%, 70.1%) |
| Asian/Pacific Islander | 0.20  (0.02, 2.28) | 1.02  (0.97, 1.08) | 0.21  (0.02, 2.33) | -0.6%  (-2.7%, 1.6%) |  | 1.21  (0.57, 2.60) | 1.00  (0.98, 1.02) | 1.22  (0.57, 2.61) | 1.2%  (-10.9%, 13.2%) |

^a^ Adjusted for maternal age (years), pre-pregnancy BMI (kg/m^2^), parity (0, 1, 2+), education level (<college degree, some college/undergraduate, graduate/post-graduate), marital status (married or living with partner, not married), family income during last year (<$30,000, $30,000-$49,999, $50,000-$99,999, $100,000 or more, not reported), plasma cotinine level (ng/mL), plasma total lipids (ng/mL), total activity (MET hours per week), sedentary activity (MET hours per week), and acculturation (US-born, recent immigrant, long-term immigrant). Observations with missing covariates were excluded from the adjusted models.

^b^ Standard errors for calculating the 95% CIs obtained using the delta method.

# **Supplemental Table S5.** Estimates of natural direct and indirect effects mediated through a WQS chemical exposure index of PBDEs for the associations of race and ethnicity with the absolute risk of PTB (on the difference scale).

| **Race and ethnicity** | **Adjusted^a^ RD (95% CI^b^) for PTB** | | | | | | |
| --- | --- | --- | --- | --- | --- | --- | --- |
|  | **Natural Direct Effect (RD_NDE_)** | **Natural Indirect Effect (RD_NIE_)** | **Controlled Direct Effects (CDEs),**  **Fixing the WQS index at the 25^th^, 50^th^ and 75^th^ percentile** | | | **Total Effect**  **(RD_TE_)** | **Proportion Mediated**  **(PM), %** |
|  |  |  | **RD_CDE(25th)_** | **RD_CDE(median)_** | **RD_CDE(75th)_** |  |  |
| Non-Hispanic White | *REF* | *REF* | *REF* | *REF* | *REF* | *REF* | *REF* |
| Non-Hispanic Black | 3.85%  (0.58, 7.5)% | 0.10%  (-0.52, 0.6)% | 3.70%  (-0.41, 7.6)% | 3.79%  (0.20, 7.6)% | 3.95%  (1.02, 8.1)% | 3.95%  (0.66, 7.7)% | 2.6%  (-20.1%, 22.5%) |
| Hispanic | -0.59%  (-3.70, 2.5)% | 0.05%  (-0.15, 0.3)% | -0.73%  (-4.91, 2.8)% | -0.64%  (-4.08, 2.5)% | -0.50%  (-3.26, 2.9)% | -0.55%  (-3.61, 2.6)% | -8.4%  (-65.0%, 78.4%) |
| Asian/Pacific Islander | -0.32%  (-3.84, 3.3)% | 0.16%  (-0.20, 1.1)% | -1.14%  (-5.53, 2.5)% | -0.63%  (-4.26, 2.5)% | 0.25%  (-3.81, 4.8)% | -0.16%  (-3.79, 3.7)% | -105.8%  (-132.7%, 130.4%) |

RD, risk difference.

^a^ Adjusted for maternal age (years), pre-pregnancy BMI (kg/m^2^), parity (0, 1, 2+), education level (<college degree, some college/undergraduate, graduate/post-graduate), marital status (married or living with partner, not married), family income during last year (<$30,000, $30,000-$49,999, $50,000-$99,999, $100,000 or more, not reported), plasma cotinine level (ng/mL), plasma total lipids (ng/mL), total activity (MET hours per week), sedentary activity (MET hours per week), and acculturation (US-born, recent immigrant, long-term immigrant). Observations with missing covariates were excluded from the adjusted models.

^b^ Standard errors for calculating the 95% CIs obtained from bootstrap method using the g-formula approach.

# **Supplemental Table S6.** Estimates of direct and indirect effects mediated through a WQS chemical exposure index of PBDEs for the associations of race and ethnicity with gestational age at delivery and preterm birth, using the g-formula approach allowing for mediator-outcome confounders affected by the exposure.

| **Race and ethnicity** | **Adjusted^a, b^ β (95% CI^c^) for gestational age at delivery, weeks** | | | | | | |
| --- | --- | --- | --- | --- | --- | --- | --- |
|  | **Natural Direct Effect (β_NDE_)** | **Natural Indirect Effect (β_NIE_)** | **Controlled Direct Effects (CDEs),**  **fixing the WQS index at the 25^th^ percentile, median, and 75^th^ percentile** | | | **Total Effect**  **(β_TE_)** | **Proportion Mediated**  **(PM), %** |
|  |  |  | **β_CDE(25th)_** | **β_CDE(median)_** | **β_CDE(75th)_** |  |  |
| Non-Hispanic White | *REF* | *REF* | *REF* | *REF* | *REF* | *REF* | *REF* |
| Non-Hispanic Black | -0.35  (-0.55, -0.11) | -0.04  (-0.10, 0.00) | -0.27  (-0.52, -0.03) | -0.32  (-0.53, -0.10) | -0.40  (-0.65, -0.15) | -0.39  (-0.60, -0.13) | 10.6%  (-1.4%, 28.9%) |
| Hispanic | -0.02  (-0.23, 0.22) | -0.02  (-0.05, 0.00) | 0.03  (-0.20, 0.31) | 0.00  (-0.22, 0.25) | -0.06  (-0.29, 0.17) | -0.04  (-0.25, 0.21) | 48.9%  (-222.1%, 388.8%) |
| Asian/Pacific Islander | -0.06  (-0.32, 0.18) | -0.01  (-0.04, 0.01) | -0.03  (-0.32, 0.24) | -0.05  (-0.32, 0.20) | -0.09  (-0.36, 0.15) | -0.07  (-0.33, 0.18) | 13.6%  (-111.7%, 81.2%) |
|  | **Adjusted^a,b^ OR (95% CI^c^) for preterm birth** | | | | | | |
|  | **Natural Direct Effect (OR_NDE_)** | **Natural Indirect Effect (OR_NIE_)** | **Controlled Direct Effects (CDEs),**  **Fixing the WQS index at the 25^th^ percentile, median, and 75^th^ percentile** | | | **Total Effect**  **(OR_TE_)** | **Proportion Mediated**  **(PM), %** |
|  |  |  | **OR_CDE(25th)_** | **OR_CDE(median)_** | **OR_CDE(75th)_** |  |  |
| Non-Hispanic White | *REF* | *REF* | *REF* | *REF* | *REF* | *REF* | *REF* |
| Non-Hispanic Black | 1.89  (1.02, 3.31) | 1.01  (0.94, 1.07) | 1.90  (1.00, 3.42) | 1.90  (1.00, 3.46) | 1.88  (1.05, 3.65) | 1.92  (1.05, 3.33) | 2.7%  (-18.1%, 25.3%) |
| Hispanic | 0.97  (0.53, 1.82) | 1.02  (0.94, 1.09) | 0.95  (0.48, 2.01) | 0.96  (0.51, 1.82) | 0.98  (0.52, 1.88) | 0.98  (0.52, 1.84) | -82.9%  (-89.9%, 565.6%) |
| Asian/Pacific Islander | 1.03  (0.57, 2.38) | 1.03  (0.86, 1.11) | 0.92  (0.45, 2.39) | 0.97  (0.45, 2.13) | 1.08  (0.27, 2.14) | 1.06  (0.62, 2.37) | 51.5%  (-253.8%, 194.4%) |

^a^ Adjusted for maternal age (years) and acculturation (US-born, recent immigrant, long-term immigrant) as base mediator-outcome confounders not affected by the exposure.

^b^ Adjusted for pre-pregnancy BMI (kg/m^2^), parity (0, 1, 2+), education level (<college degree, some college/undergraduate, graduate/post-graduate), marital status (married or living with partner, not married), family income during last year (<$30,000, $30,000-$49,999, $50,000-$99,999, $100,000 or more, not reported), plasma cotinine level (ng/mL), plasma total lipids (ng/mL), total activity (MET hours per week) and sedentary activity (MET hours per week) as mediator-outcome confounders potentially affected by the exposure.

^c^ Effect estimates were obtained from direct counterfactual imputation estimation. The standard errors of causal effects are obtained by the standard deviations of bootstrapped results.

# **Supplemental Table S7.** Estimates of direct and indirect effects mediated through a binary WQS chemical exposure index of PBDEs (WQS ≥ median vs. WQS < median) for the associations of race and ethnicity with gestational age at delivery and preterm birth.

| **Race and ethnicity** | **Adjusted^a^ β (95% CI^b^) for gestational age at delivery, weeks** | | | | | |
| --- | --- | --- | --- | --- | --- | --- |
|  | **Natural Direct Effect (β_NDE_)** | **Natural Indirect Effect (β_NIE_)** | **Controlled Direct Effects (CDEs)** | | **Total Effect**  **(β_TE_)** | **Proportion Mediated**  **(PM), %** |
|  |  |  | **β_CDE(WQS < median )_** | **β_CDE(WQS ≥ median)_** |  |  |
| Non-Hispanic White | *REF* | *REF* | *REF* | *REF* | *REF* | *REF* |
| Non-Hispanic Black | -0.26  (-0.51, -0.01) | -0.03  (-0.07, 0.01) | -0.09  (-0.41, 0.24) | -0.47  (-0.79, -0.15) | -0.30  (-0.54, -0.05) | 11.3%  (-5.1%, 27.7%) |
| Hispanic | 0.05  (-0.20, 0.30) | -0.01  (-0.03, 0.01) | 0.10  (-0.21, 0.41) | -0.01  (-0.34, 0.31) | 0.04  (-0.21, 0.29) | -18.7%  (-142.6%, 105.1%) |
| Asian/Pacific Islander | -0.06  (-0.32, 0.20) | -0.00  (-0.01, 0.01) | -0.07  (-0.40, 0.26) | -0.04  (-0.40, 0.32) | -0.06  (-0.32, 0.20) | 0.06%  (-24.4%, 24.5%) |
|  | **Adjusted^a^ OR (95% CI^b^) for preterm birth** | | | | | |
|  | **Natural Direct Effect (OR_NDE_)** | **Natural Indirect Effect (OR_NIE_)** | **Controlled Direct Effects (CDEs)** | | **Total Effect**  **(OR_TE_)** | **Proportion Mediated**  **(PM), %** |
|  |  |  | **OR_CDE(WQS < median)_** | **OR_CDE(WQS ≥ median)_** |  |  |
| Non-Hispanic White | *REF* | *REF* | *REF* | *REF* | *REF* | *REF* |
| Non-Hispanic Black | 1.79  (0.98, 3.27) | 1.02  (0.96, 1.07) | 1.74  (0.78, 3.91) | 1.85  (0.85, 4.00) | 1.82  (1.00, 3.32) | 3.5%  (-8.7%, 15.6%) |
| Hispanic | 0.90  (0.45, 1.77) | 1.01  (0.97, 1.06) | 0.86  (0.35, 2.14) | 0.93  (0.39, 2.21) | 0.91  (0.46, 1.79) | -11.3%  (-104.5%, 82.0%) |
| Asian/Pacific Islander | 0.96  (0.46, 2.00) | 0.99  (0.94, 1.04) | 1.17  (0.46, 2.94) | 0.74  (0.26, 2.10) | 0.95  (0.46, 1.97) | 22.0%  (-293.3%, 337.3%) |

^a^ Adjusted for maternal age (years), pre-pregnancy BMI (kg/m^2^), parity (0, 1, 2+), education level (<college degree, some college/undergraduate, graduate/post-graduate), marital status (married or living with partner, not married), family income during last year (<$30,000, $30,000-$49,999, $50,000-$99,999, $100,000 or more, not reported), plasma cotinine level (ng/mL), plasma total lipids (ng/mL), total activity (MET hours per week), sedentary activity (MET hours per week), and acculturation (US-born, recent immigrant, long-term immigrant). Observations with missing covariates were excluded from the adjusted models.

^b^ Standard errors for calculating the 95% CIs obtained using the delta method.

# **Supplemental Table S8.** Associations between levels of plasma PBDEs concentrations and WQS index (as quantiles) and gestational age at delivery.

| **Plasma PBDEs** | **Population marginal means of gestational age at delivery in weeks (95% CI)^a, b^** | **Plasma PBDEs (continued)** | **Population marginal means of gestational age at delivery in weeks (95% CI)^a, b^** |
| --- | --- | --- | --- |
| **PBDE 28 ^a, b^** | | **PBDE 153 ^a, b^** |  |
| Below LOQ | 39.51 (39.20, 39.82) | Below LOQ | 39.57 (39.27, 39.88) |
| Q1 above LOQ | 39.66 (39.28, 40.04) | Q1 above LOQ | 39.72 (39.33, 40.11) |
| Q2 above LOQ | 39.62 (39.24, 40.00) | Q2 above LOQ | 39.55 (39.18, 39.92) |
| Q3 above LOQ | 39.51 (39.13, 39.89) | Q3 above LOQ | 39.40 (39.03, 39.77) |
| Q4 above LOQ | 39.40 (39.00, 39.80) | Q4 above LOQ | 39.23 (38.85, 39.61)^d^ |
| p-trend^c^ | 0.74 | p-trend^c^ | 0.01 |
|  |  |  |  |
| **PBDE 47 ^a, b^** | | **PBDE 154 ^a, b^** |  |
| Below LOQ | 39.62 (39.20, 40.03) | Below LOQ | 39.45 (39.15, 39.76) |
| Q1 above LOQ | 39.51 (39.17, 39.85) | Q1 above LOQ | 39.63 (39.25, 40.00) |
| Q2 above LOQ | 39.43 (39.10, 39.76) | Q2 above LOQ | 39.66 (39.28, 40.04) |
| Q3 above LOQ | 39.49 (39.16, 39.82) | Q3 above LOQ | 39.65 (39.27, 40.02) |
| Q4 above LOQ | 39.61 (39.26, 39.96) | Q4 above LOQ | 39.58 (39.20, 39.95) |
| p-trend^c^ | 0.53 | p-trend^c^ | 0.09 |
|  |  |  |  |
| **PBDE 99 ^a, b^** | | **WQS index ^a^** |  |
| Below LOQ | 39.53 (39.21, 39.84) | Q1 | 39.51 (39.22, 39.79) |
| Q1 above LOQ | 39.59 (39.25, 39.94) | Q2 | 39.39 (39.11, 39.67) |
| Q2 above LOQ | 39.38 (39.04, 39.72) | Q3 | 39.56 (39.29, 39.84) |
| Q3 above LOQ | 39.54 (39.20, 39.89) | Q4 | 39.30 (39.03, 39.58)^d^ |
| Q4 above LOQ | 39.51 (39.14, 39.88) | Q5 | 39.28 (39.01, 39.56)^d^ |
| p-trend^c^ | 0.85 | p-trend^c^ | 0.04 |
|  |  |  |  |
| **PBDE 100 ^a, b^** | |  |  |
| Below LOQ | 39.50 (39.17, 39.83) |  |  |
| Q1 above LOQ | 39.53 (39.18, 39.87) |  |  |
| Q2 above LOQ | 39.49 (39.15, 39.83) |  |  |
| Q3 above LOQ | 39.43 (39.09, 39.77) |  |  |
| Q4 above LOQ | 39.62 (39.25, 40.00) |  |  |
| p-trend^c^ | 0.57 |  |  |

SD = standard deviation; CI = confidence interval.

^a^ Adjusted for maternal age (years), race/ethnicity (Non-Hispanic White, Non-Hispanic Black, Hispanic, Asian and Pacific Islander), pre-pregnancy BMI (kg/m^2^), parity (0, 1, 2+), education level (<college degree, some college/undergraduate, graduate/post-graduate), marital status (married or living with partner, not married), income during last year (Less than $30,000, $30,000-$49,999, $50,000-$99,999, $100,000 or more, not reported), plasma cotinine level (ng/mL), plasma total lipids (ng/mL), total activity (MET hours per week), sedentary activity (MET hours per week) and acculturation (US-born, recent immigrant, long-term immigrant).

^b^ Additionally adjusted for all the other PBDEs concentrations as continuous covariates.

^c^ p-trend obtained from fitting median concentrations of each category as continuous exposures.

^d^ p-value < 0.05 compared to the lowest category.

# **Supplemental Table S9.** Estimates of direct and indirect effects mediated through the WQS index of PBDEs (WQS as quintiles) for the associations of race and ethnicity with gestational age at delivery and preterm birth.

| **Race and ethnicity** | **Adjusted^a^ β (95% CI^b^) for gestational age at delivery, weeks** | | | | | | | | |
| --- | --- | --- | --- | --- | --- | --- | --- | --- | --- |
|  | **Natural Direct Effect (β_NDE_)** | **Natural Indirect Effect (β_NIE_)** | **Controlled Direct Effects (CDEs)** | | | | | **Total Effect**  **(β_TE_)** | **Proportion Mediated**  **(PM), %** |
|  |  |  | **β_CDE(Q1)_** | **β_CDE(Q2)_** | **β_CDE(Q3)_** | **β_CDE(Q4)_** | **β_CDE(Q5)_** |  |  |
| Non-Hispanic White | *REF* | *REF* | *REF* | *REF* | *REF* | *REF* | *REF* | *REF* | *REF* |
| Non-Hispanic Black | -0.25  (-0.49, 0.00) | -0.05  (-0.10, 0.00) | -0.02  (-0.41, 0.37) | -0.15  (-0.43, 0.14) | -0.27  (-0.51, -0.03) | -0.39  (-0.68, -0.12) | -0.52  (-0.89, -0.15) | -0.30  (-0.54, -0.05) | 16.6%  (-4.2%, 37.4%) |
| Hispanic | 0.06  (-0.19, 0.31) | -0.00  (-0.03, 0.02) | 0.13  (-0.23, 0.50) | 0.09  (-0.19, 0.37) | 0.04  (-0.20, 0.29) | -0.00  (-0.29, 0.29) | -0.05  (-0.42, 0.33) | 0.06  (-0.19, 0.31) | -38.4%  (-299.4%, 222.6%) |
| Asian/Pacific Islander | -0.05  (-0.31, 0.20) | -0.01  (-0.03, 0.02) | -0.08  (-0.47, 0.31) | -0.07  (-0.35, 0.23) | -0.05  (-0.31, 0.21) | -0.04  (-0.35, 0.28) | -0.02  (-0.45, 0.40) | -0.06  (-0.32, 0.20) | 2.2%  (-42.5%, 46.9%) |
|  | **Adjusted^a^ OR (95% CI^b^) for preterm birth** | | | | | | | | |
|  | **Natural Direct Effect (OR_NDE_)** | **Natural Indirect Effect (OR_NIE_)** | **Controlled Direct Effects (CDEs)** | | | | | **Total Effect**  **(OR_TE_)** | **Proportion Mediated**  **(PM), %** |
|  |  |  | **OR_CDE(Q1)_** | **OR_CDE(Q2)_** | **OR_CDE(Q3)_** | **OR_CDE(Q4)_** | **OR_CDE(Q5)_** |  |  |
| Non-Hispanic White | *REF* | *REF* | *REF* | *REF* | *REF* | *REF* | *REF* | *REF* | *REF* |
| Non-Hispanic Black | 1.80  (0.99, 3.31) | 1.01  (0.93, 1.10) | 1.90  (0.73, 4.98) | 1.85  (0.90, 3.80) | 1.80  (0.99, 3.31) | 1.75  (0.89, 3.46) | 1.71  (0.69, 4.22) | 1.82  (1.00, 3.31) | 2.7%  (-14.8%, 20.1%) |
| Hispanic | 0.90  (0.45, 1.79) | 1.01  (0.94, 1.09) | 0.92  (0.31, 2.74) | 0.91  (0.40, 2.06) | 0.90  (0.46, 1.78) | 0.89  (0.41, 1.92) | 0.88  (0.32, 2.44) | 0.90  (0.46, 1.77) | -12.3%  (-136.2%, 110.2%) |
| Asian/Pacific Islander | 0.98  (0.49, 2.06) | 0.95  (0.45, 1.99) | 1.42  (0.47, 4.34) | 1.15  (0.50, 2.63) | 0.93  (0.44, 1.95) | 0.75  (0.30, 1.89) | 0.60  (0.17, 2.13) | 0.97  (0.47, 2.01) | 59.6%  (-898.8%, 1018.0%) |

WQS: weighted quantile sum. Q1-Q4: quartiles 1 to 4 (above LOQ).

^a^ Adjusted for maternal age (years), pre-pregnancy BMI (kg/m^2^), parity (0, 1, 2+), education level (<college degree, some college/undergraduate, graduate/post-graduate), marital status (married or living with partner, not married), family income during last year (<$30,000, $30,000-$49,999, $50,000-$99,999, $100,000 or more, not reported), plasma cotinine level (ng/mL), plasma total lipids (ng/mL), total activity (MET hours per week), sedentary activity (MET hours per week), and acculturation (US-born, recent immigrant, long-term immigrant). ^b^ Standard errors for calculating the 95% CIs obtained using the delta method, based on point estimates obtained using closed-form parameter function estimation.

# **Supplemental Table S10.** Estimates of direct and indirect effects mediated through a PBDE 153 as quantiles for the associations of race and ethnicity with gestational age at delivery and preterm birth.

| **Race and ethnicity** | **Adjusted^a^ β (95% CI^b^) for gestational age at delivery, weeks** | | | | | | | | |
| --- | --- | --- | --- | --- | --- | --- | --- | --- | --- |
|  | **Natural Direct Effect (β_NDE_)** | **Natural Indirect Effect (β_NIE_)** | **Controlled Direct Effects (CDEs)** | | | | | **Total Effect**  **(β_TE_)** | **Proportion Mediated**  **(PM), %** |
|  |  |  | **β_CDE(<LOQ)_** | **β_CDE(Q1 above LOQ)_** | **β_CDE(Q2 above LOQ)_** | **β_CDE(Q3 above LOQ)_** | **β_CDE(Q4 above LOQ)_** |  |  |
| Non-Hispanic White | *REF* | *REF* | *REF* | *REF* | *REF* | *REF* | *REF* | *REF* | *REF* |
| Non-Hispanic Black | -0.28  (-0.51, -0.03) | -0.03  (-0.08, 0.00) | -0.21  (-0.48, 0.09) | -0.14  (-0.92, 0.54) | -0.45  (-1.53, 0.45) | -0.55  (-1.25, 0.02) | -0.43  (-1.08, 0.26) | -0.30  (-0.53, -0.06) | 9.3%  (-1.5%, 41.7%) |
| Hispanic | 0.03  (-0.17, 0.24) | -0.00  (-0.02, 0.01) | 0.04  (-0.24, 0.26) | 0.29  (-0.28, 1.01) | -0.08  (-0.74, 0.55) | -0.19  (-0.81, 0.38) | 0.05  (-0.51, 0.58) | 0.03  (-0.17, 0.23) | -5.3%  (-34.7%, 123.8%) |
| Asian/Pacific Islander | -0.08  (-0.35, 0.13) | 0.00  (-0.01, 0.02) | -0.15  (-0.50, 0.17) | 0.05  (-0.67, 0.77) | 0.28  (-0.33, 0.91) | 0.21  (-0.62, 0.92) | -0.37  (-0.95, 0.26) | -0.07  (-0.35, 0.12) | 6.9%  (-85.6%, 51.6%) |
|  | **Adjusted^a^ OR (95% CI^b^) for preterm birth** | | | | | | | | |
|  | **Natural Direct Effect (OR_NDE_)** | **Natural Indirect Effect (OR_NIE_)** | **Controlled Direct Effects (CDEs)** | | | | | **Total Effect**  **(OR_TE_)** | **Proportion Mediated**  **(PM), %** |
|  |  |  | **OR_CDE(<LOQ)_** | **OR_CDE(Q1 above LOQ)_** | **OR_CDE(Q2 above LOQ)_** | **OR_CDE(Q3 above LOQ)_** | **OR_CDE(Q4 above LOQ)_** |  |  |
| Non-Hispanic White | *REF* | *REF* | *REF* | *REF* | *REF* | *REF* | *REF* | *REF* | *REF* |
| Non-Hispanic Black | 1.80  (0.96, 3.05) | 0.98  (0.91, 1.06) | 2.14  (0.94, 5.59) | 1.80  (0.35, 16.5) | 1.80  (0.38, 12.7) | 1.52  (0.37, 8.18) | 0.80  (0.21, 3.08) | 1.77  (0.97, 3.07) | 3.6%  (-43.5%, 54.0%) |
| Hispanic | 0.91  (0.48, 1.67) | 1.00  (0.96 1.05) | 1.12  (0.48, 2.21) | 2.01  (0.00, 1.74) | 0.89  (0.00, 54.0) | 0.82  (0.00, 7.92) | 0.74  (0.00, 4.11) | 0.90  (0.48, 1.72) | 3.3%  (-115.5%, 51.8%) |
| Asian/Pacific Islander | 1.01  (0.38, 2.08) | 1.01  (0.93, 1.08) | 1.14  (0.35, 2.95) | 1.21  (0.00, 138.1) | /  (non-converge) | 0.61  (0.00, 3.32) | 1.41  (0.00, 6.72) | 1.03  (0.38, 2.08) | 58.1%  (-508.8%, 102.8%) |

LOQ: limits of quantification. Q1-Q4: quartiles 1 to 4 (above LOQ).

PBDE 153 was included in the model as an ordinal mediator: <LOQ, Q1 above LOQ, Q2 above LOQ, Q3 above LOQ, and Q4 above LOQ.

^a^ Adjusted for maternal age (years), pre-pregnancy BMI (kg/m^2^), parity (0, 1, 2+), education level (<college degree, some college/undergraduate, graduate/post-graduate), marital status (married or living with partner, not married), family income during last year (<$30,000, $30,000-$49,999, $50,000-$99,999, $100,000 or more, not reported), plasma cotinine level (ng/mL), plasma total lipids (ng/mL), total activity (MET hours per week), sedentary activity (MET hours per week), and acculturation (US-born, recent immigrant, long-term immigrant). ^b^ Effect estimates were obtained from direct counterfactual imputation estimation which accommodates an ordinal mediator (i.e., fitting an ordinal logistic model for the mediator model). The standard errors of causal effects are obtained by the standard deviations of bootstrapped results.

# **Supplemental Table S11.** Estimates of direct and indirect effects mediated through a weighted exposure index of 4 PBDEs (PBDE 28, 99, 100, 153, selected from quantile g-computation with weights towards an inverse association between PBDEs and gestational age at delivery) for the associations of race and ethnicity with gestational age at delivery and preterm birth.

| **Race and ethnicity** | **Adjusted^a^ β (95% CI^b^) for gestational age at delivery, weeks** | | | | | | |
| --- | --- | --- | --- | --- | --- | --- | --- |
|  | **Natural Direct Effect (β_NDE_)** | **Natural Indirect Effect (β_NIE_)** | **Controlled Direct Effects (CDEs),**  **fixing the index at the 25^th^ percentile, median, and 75^th^ percentile** | | | **Total Effect**  **(β_TE_)** | **Proportion Mediated**  **(PM), %** |
|  |  |  | **β_CDE(25th)_** | **β_CDE(median)_** | **β_CDE(75th)_** |  |  |
| Non-Hispanic White | *REF* | *REF* | *REF* | *REF* | *REF* | *REF* | *REF* |
| Non-Hispanic Black | -0.25  (-0.50, 0.00) | -0.04  (-0.08, -0.00) | -0.15  (-0.44, 0.13) | -0.21  (-0.47, 0.05) | -0.30  (-0.55, -0.06) | -0.30  (-0.54, -0.05) | 14.8%  (-3.4%, 33.1%) |
| Hispanic | 0.07  (-0.18, 0.32) | -0.01  (-0.03, 0.01) | 0.11  (-0.18, 0.39) | 0.08  (-0.18, 0.34) | 0.04  (-0.21, 0.30) | 0.06  (-0.19, 0.31) | -11.3%  (-70.8%, 48.1%) |
| Asian/Pacific Islander | -0.06  (-0.32, 0.21) | -0.01  (-0.02, 0.01) | -0.02  (-0.33, 0.28) | -0.04  (-0.31, 0.23) | -0.07  (-0.34, 0.20) | -0.06  (-0.32, 0.20) | 9.6%  (-40.7%, 59.9%) |
|  | **Adjusted^a^ OR (95% CI^b^) for preterm birth** | | | | | | |
|  | **Natural Direct Effect (OR_NDE_)** | **Natural Indirect Effect (OR_NIE_)** | **Controlled Direct Effects (CDEs),**  **Fixing the index at the 25^th^ percentile, median, and 75^th^ percentile** | | | **Total Effect**  **(OR_TE_)** | **Proportion Mediated**  **(PM), %** |
|  |  |  | **OR_CDE(25th)_** | **OR_CDE(median)_** | **OR_CDE(75th)_** |  |  |
| Non-Hispanic White | *REF* | *REF* | *REF* | *REF* | *REF* | *REF* | *REF* |
| Non-Hispanic Black | 1.78  (0.97, 3.25) | 1.01  (0.96, 1.07) | 1.80  (0.95, 3.40) | 1.80  (0.95, 3.40) | 1.76  (0.97, 3.21) | 1.82  (1.00, 3.31) | 2.7%  (-9.6%, 15.1%) |
| Hispanic | 0.87  (0.43, 1.70) | 1.01  (0.97, 1.04) | 0.85  (0.38, 1.90) | 0.85  (0.42, 1.76) | 0.86  (0.43, 1.72) | 0.90  (0.46, 1.77) | -4.1%  (-33.9%, 25.6%) |
| Asian/Pacific Islander | 0.96  (0.47, 1.97) | 1.02  (0.96, 1.08) | 0.79  (0.33, 1.85) | 0.85  (0.39, 1.86) | 0.99  (0.48, 2.03) | 0.97  (0.47, 2.01) | -99.2%  (-3531.9%, 3333.4%) |

^a^ Adjusted for maternal age (years), pre-pregnancy BMI (kg/m^2^), parity (0, 1, 2+), education level (<college degree, some college/undergraduate, graduate/post-graduate), marital status (married or living with partner, not married), family income during last year (<$30,000, $30,000-$49,999, $50,000-$99,999, $100,000 or more, not reported), plasma cotinine level (ng/mL), plasma total lipids (ng/mL), total activity (MET hours per week), sedentary activity (MET hours per week), and acculturation (US-born, recent immigrant, long-term immigrant). Observations with missing covariates were excluded from the adjusted models.

^b^ Standard errors for calculating the 95% CIs obtained using the delta method, based on point estimates obtained using closed-form parameter function estimation.


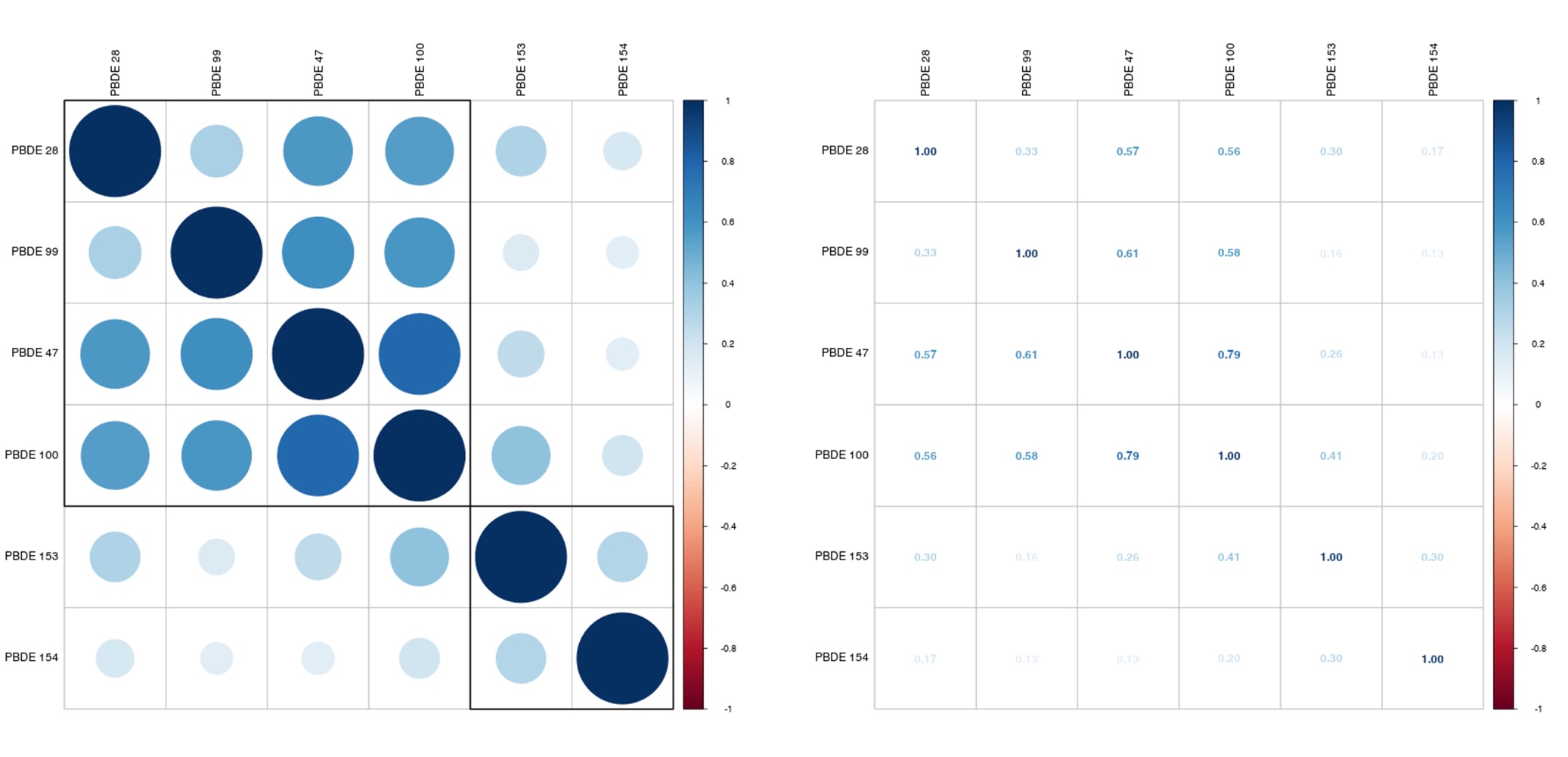


# **Supplemental Figure S1.** Correlation plot of the concentrations of PBDEs chemicals in this study.

Dot size (left figure) was proportional to the magnitude of Spearman correlation coefficients (right figure). Rectangles around the plot of correlation matrix were based on the results of hierarchical clustering.


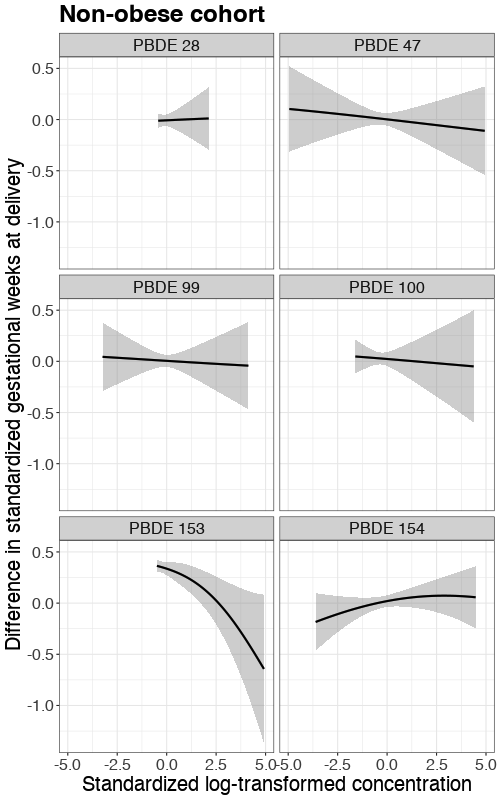


# **Supplemental Figure S2.** Univariate exposure-response functions (95% credible intervals) between PBDE concentrations and standardized gestational age at delivery estimated by BKMR with hierarchical variable selection, holding all other congeners at the median levels, adjusted for confounders.


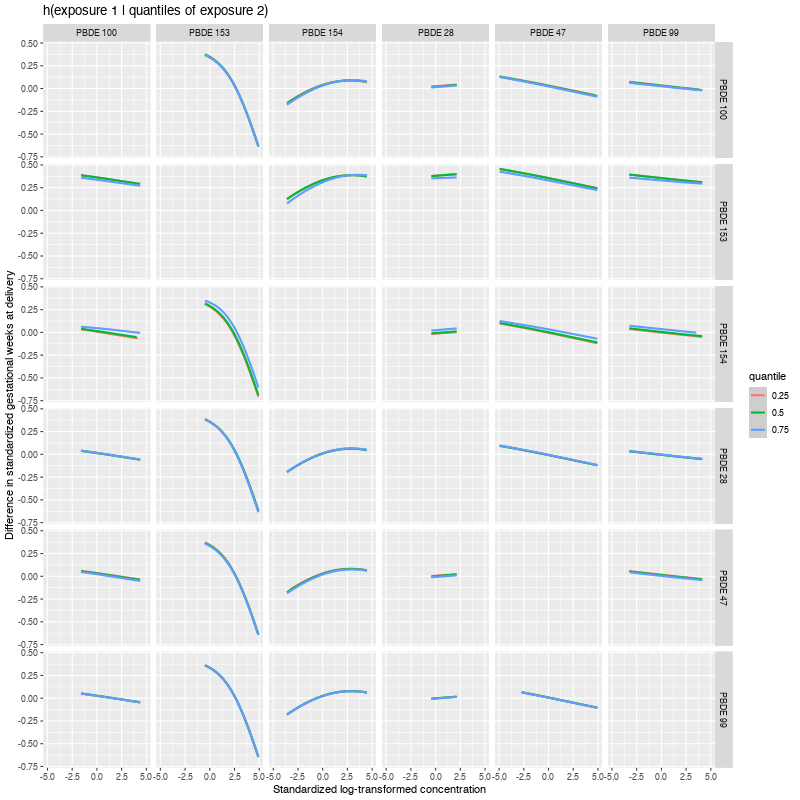


# **Supplemental Figure S3.** Exposure-response functions between each chemical and gestational age at delivery, when fixing another chemical’s concentrations at the 25th, 50th, and 75th percentiles, and all other congeners at the median, estimated from BKMR with hierarchical variable selection.


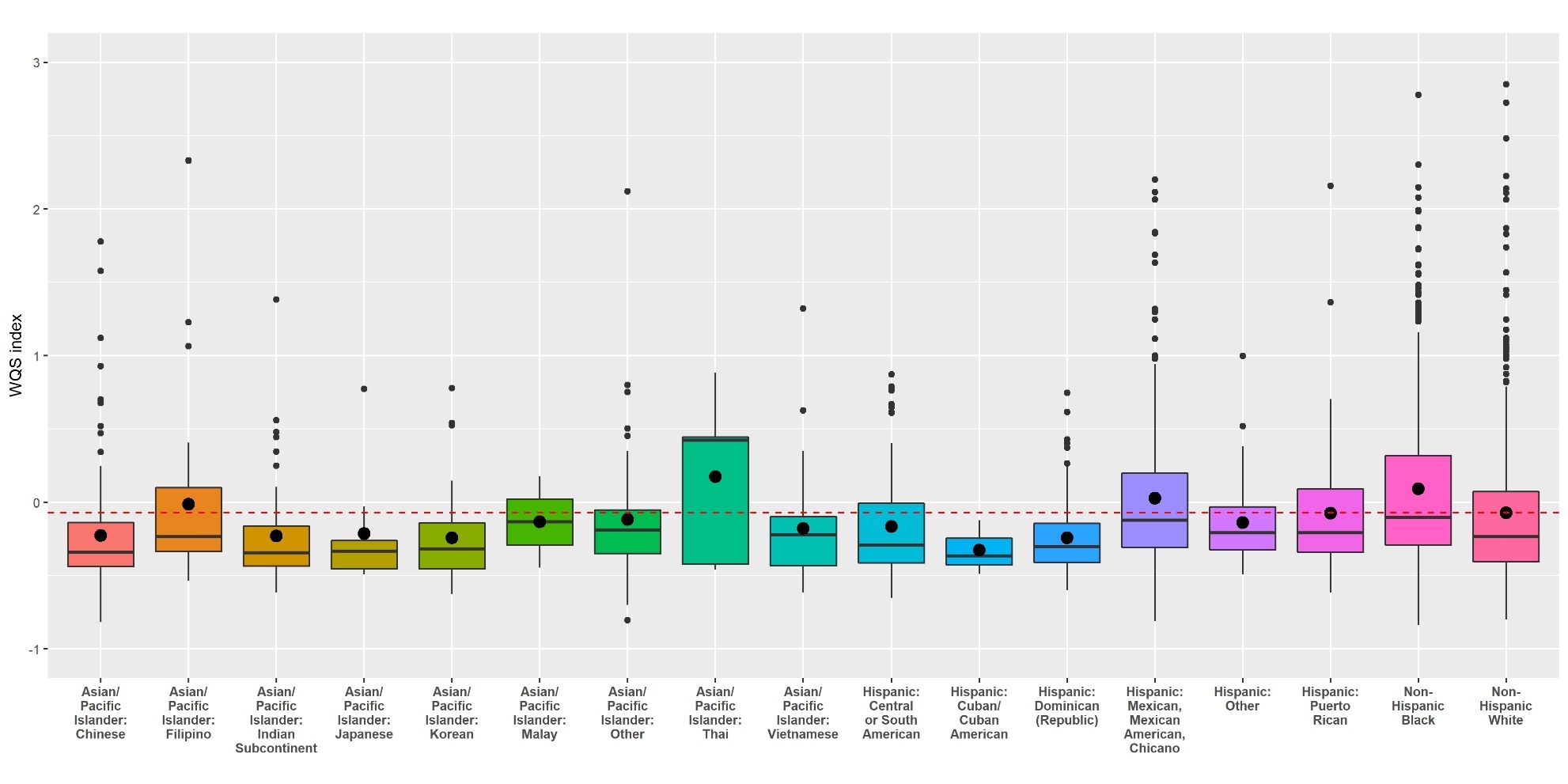


# **Supplemental Figure S4.** Box plot of the WQS index of PBDEs stratified by race and ethnicity, including Hispanic origin or Asian background.

The dot within each box represents the mean value of the WQS index. The red dashed line represents the mean value of the WQS index among non-Hispanic White women. Other visualizing components can be interpreted as conventional box plots.


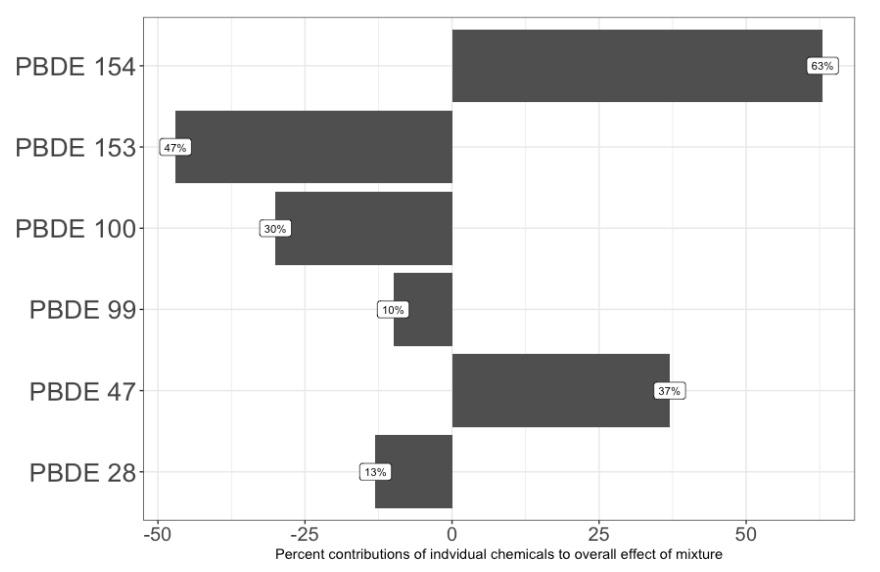


**Supplemental Figure S5**. Weights of PBDEs estimated from quantile g-computation.

Chemicals contributing to an inverse association between PBDEs and gestational age at delivery (PBDE 28, 99, 100, and 153) were included in the sensitivity analysis using a weighted index of these four chemicals as a mediator.


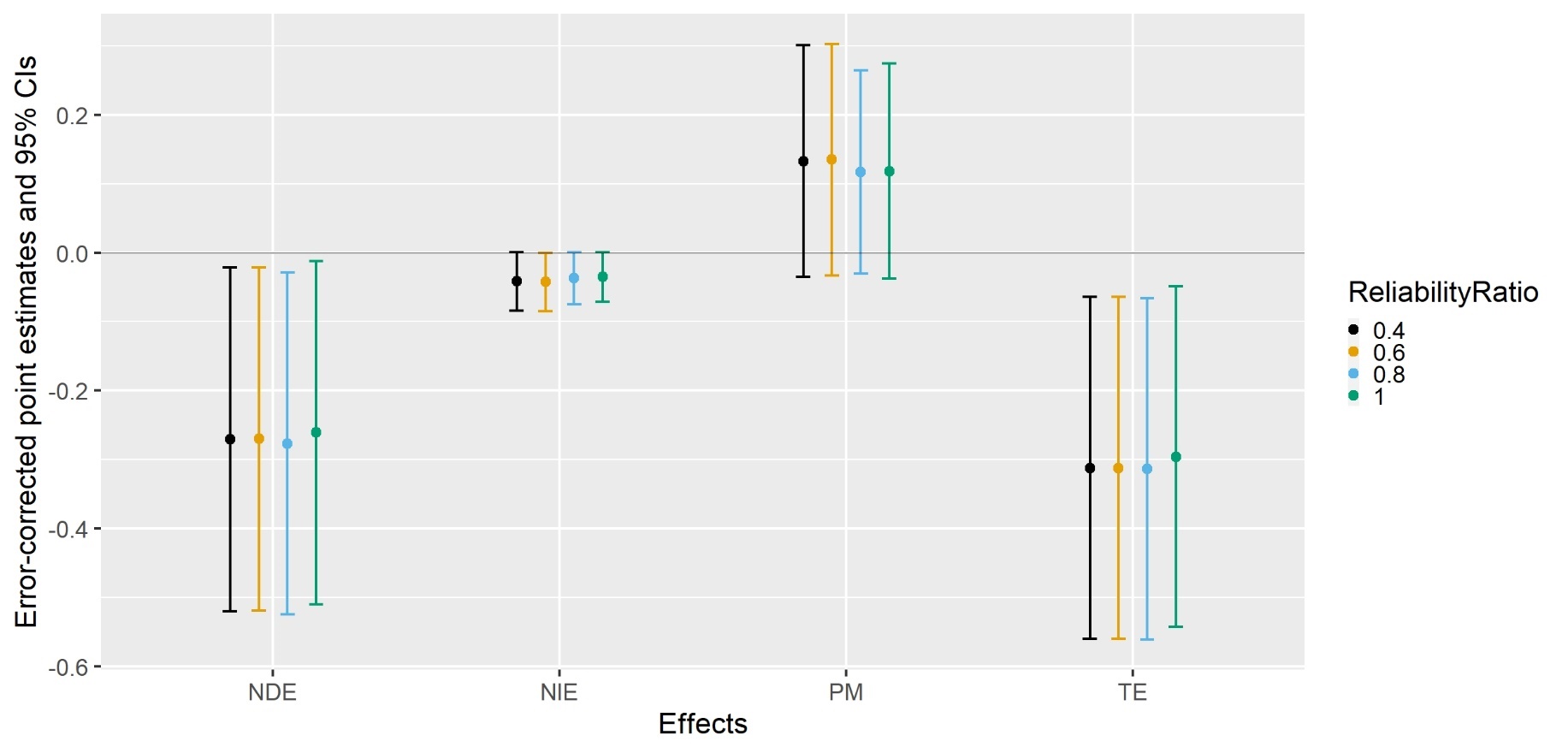


# **Supplemental Figure S6.** Summary of error-corrected effect estimates for the mediation analysis comparing non-Hispanic Black to non-Hispanic White women on gestational age at delivery, assuming different levels of measurement error in the mediator (i.e. WQS).

NDE: natural direct effect; NIE: natural indirect effect; PM: proportion mediated; TE: total effect. Point estimates and 95% CIs in weeks for NDE, NIE and TE.

Measurement error correction conducted using the SIMEX method with default 200 simulations.

Reliability ratios calculated by 1 - MEerror[i]/sd(data[, MEvariable]), where MEerror is a vector of standard deviations of the measurement error, and sd(data[, MEvariable]) is the standard deviation of the variable with measurement error.
